# The role of the polymeric network in the water sensitivity of modern oil paints

**DOI:** 10.1038/s41598-019-39963-z

**Published:** 2019-03-05

**Authors:** Jacopo La Nasa, Judith Lee, Ilaria Degano, Aviva Burnstock, Klaas Jan van den Berg, Bronwyn Ormsby, Ilaria Bonaduce

**Affiliations:** 1 Department of Chemistry and Industrial Chemistry, University of Pisa, via Moruzzi, 13-56124 Pisa, Italy; 2 Conservation Department, Tate, Millbank, London, SW1P 4RG United Kingdom; 3 Courtauld Institute of Art, Somerset House, Strand, London, WC2R 0RN United Kingdom; 4 Cultural Heritage Agency of the Netherlands (RCE), Hobbemastraat 22, 1071 Amsterdam, Netherlands

## Abstract

Spectroscopic and mass spectrometric analytical techniques were used to characterise two naturally aged Winsor & Newton (W&N) Winsor Green (phthalocyanine green, PG7) artists’ oil colour paint swatches dating to 1993 and 2003. Infrared and Energy Dispersive X-ray (EDX) analysis indicated that the swatches were of closely similar composition, yet the swatch from 2003 was water-sensitive whilst the swatch from 1993 was not. Water-sensitivity is a conservation challenge associated with significant numbers of modern oil paintings and this study aimed to further develop our understanding of the molecular causes of water sensitivity. SEM elemental mapping of samples taken from both swatches provided no indication for the formation of epsomite – a known cause of water sensitivity in some modern oil paintings. Liquid chromatography coupled with mass spectrometry (HPLC-MS) and gas chromatography coupled with mass spectrometry (GC-MS) also revealed very similar qualitative-quantitative composition in terms of unbound and esterified medium fractions. The polymeric network was investigated using analytical pyrolysis. A combination of flash pyrolysis coupled with gas chromatography mass spectrometry (Py-GC-MS) together with evolved gas analysis mass spectrometry (EGA-MS) revealed that the polymeric material was relatively more abundant in the non-water-sensitive paint. This is the first multi-analytical study that has demonstrated a correlation between water-sensitivity and the degree of polymerisation of the oil medium; independent of other known causes of water-sensitivity.

## Introduction

There are many examples of unvarnished (uncoated) water sensitive twentieth and twenty-first century (modern) oil paintings, which limit or preclude the use of water or protic solvents for surface cleaning treatment and the removal of polar coatings. In these cases, the application of aqueous-based cleaning materials may result in the loss of pigment and/or binder and cause unacceptable gloss changes due to surface disruption^1^. The identification of safe and effective materials and methods for the surface cleaning of water sensitive oil paintings is an area of ongoing research, which has been directly informed by parallel studies investigating the complex causes of water sensitivity^2,3^.

Studies have demonstrated that under the influence of specific environmental conditions, the formation of epsomite (magnesium sulphate heptahydrate) is one cause of water sensitivity in artists’ oil paints that contain magnesium carbonate as an extender^1,4^. Others have explored the relationship between water sensitivity, the molecular composition of the binding medium, and various aspects of modern artists’ oil paint formulation; including the influence of pigments and metal soap additives^5–9^. The results suggest that many water-sensitive paints contain a relatively high content of extractable dicarboxylic acids in comparison to non-water sensitive paints – such as those containing zinc oxide or lead white. In specific cases, such as cobalt blue and umber containing paints, there is a tendency towards a higher degree of oxidation (i.e. a high dicarboxylic acid content) that may also relate to water-sensitivity. However it has also been demonstrated that, for paints pigmented with three pigment types (two types of cobalt blue, and red earth), differences in the *severity* of water-sensitivity of paints made with the same type of pigment did not appear to relate to: the total or free (extractable) dicarboxylic acid content, metal soaps of free (non-crosslinked) fatty and dicarboxylic acids, and the overall degree of hydrolysis of ester bonds^6^. This led to the hypothesis that the degree of crosslinking, the polarity of the polymeric network, and, depending on the pigment, the nature of the ionomeric network were likely to influence the development and severity of water-sensitivity; which has been explored further in this study.

Artists’ oil paints are manufactured using drying oils, semi-drying oils, or mixtures of the two^10^. An oil binding medium containing a relatively high number of carbon-carbon double bonds forms a crosslinked film via a complex series of autoxidative radical chain reactions^11,12^. The drying process progresses through two main, competitive pathways; one leading to the formation of a cross-linked polymeric network, and one leading to oxidative scission resulting in the formation of dicarboxylic acids, as final products of oxidation^12,13^. Autoxidation of lipids is affected by the presence of pigments, metal soap additives^14–17^, as well as environmental conditions, including oxygen availability, temperature and relative humidity^18,19^. Moreover these reactive metal ions may be also present in pigments and additives. These species may become integral part of the cross-linked network, forming ionomer-like structures in cured oil paint films.

This study investigates the relationship between the degree of polymerization in an oil paint and water sensitivity. For this study, two Winsor & Newton (W&N) Winsor Green Artists’ Oil Colour paint swatches dating from 1993 and 2003 were selected. Standardised cotton swab rolling tests were used to determine the water sensitivity of the samples, where swatch from 1993 was rated as non-water-sensitive (no pigment was picked up after 50 swab rolls), and the swatch from 2003 proved to be sensitive after 6 rolls. Optical microscopy (visible and ultraviolet (UV) light), digital microscopy (HIROX) and Environmental Scanning Electron Microscopy with Energy-Dispersive X-ray Spectroscopy (ESEM-EDX) were used to characterise the elemental composition and surface morphologies of samples taken from the swatches. These techniques, together with infrared spectroscopy (FTIR), excluded the possibility of epsomite as the cause of the water sensitivity^1,4^.

The organic soluble fraction of each sample was characterized as a lipid profile using liquid chromatography coupled with high resolution mass spectrometry (HPLC-ESI-Q-ToF), involving a new derivatization method using 2-hydrazinoquinoline^20,21^. The extractable acyl glycerides (monoglyceride, diglycerides, and triglycerides) and their relative oxidation products together with the free fatty acids, were characterised and semi-quantified in a single chromatographic run. The soluble fraction was further characterised using a new GC-MS two-step analytical approach based on the selective derivatization of free fatty and dicarboxylic acids, and the metal carboxylates of free fatty and dicarboxylic acids, achieved via derivatisation with N,O-bis(trimethylsilyl)acetamide (BSTFA) and hexamethyldisilazane (HMDS)^21,22^. This new approach enabled the quantification of free metal soaps and free dicarboxylic acids within the soluble fraction, which was compared and combined with the results obtained using HPLC-ESI-Q-ToF.

Analytical pyrolysis - a combination of flash pyrolysis coupled with gas chromatography mass spectrometry (Py-GC-MS) and Evolved Gas Analysis coupled with mass spectrometry (EGA-MS) - was used to explore the polymeric fraction of the paints and the thermal stability of the different molecular fractions of the paint layers. Table 1 summarises the analyses performed on each paint swatch and medium sample fractions.

**Table 1:** Summary of the analysed performed on the paint swatches and sample fraction characterised using each technique.

Analytical method employed	Inorganic materials	Organic materials					
		Non-polymerised fraction			Polymerised fraction		
	Pigments and additives	free fatty acids and diacids	free metal soaps of fatty acids and diacids	non-crosslinked glycerides	metal soaps of diacids that are connected to a covalently bound polymeric network, only via ester bonds	fatty acid and non-saponified diacids that are connected to a covalently bound polymeric network, only via ester bonds	cross-linked material (fatty and diacids) that would remain covalently bound (C-C and C-O-C bounds), even if hydrolysis of all ester bounds present in the binder occurred
OM	✘/✓^a^	✘/✓^a^	✘/✓^a^	✘	✘	✘	✘
SEM-EDX	✓	✘	✘	✘	✘	✘	✘
FT-IR	✓	✓	✓	✓	✓	✓	✓
HPLC-ESI-Q-ToF	✘	✓	✘	✓	✘	✘	✘
GCMS (BSTFA)	✘	✓	✓	✘	✘	✘	✘
GCMS (HMDS)	✘	✓	✘	✘	✘	✘	✘
GCMS (Meth Prep II)	✘	✓^b^	✓^b^	✓^b^	✓^b^	✓^b^	✘
Py-GC-MS^b^	✘	✓	✓	✓	✓	✓	✓
EGA-MS	✘	✓	✓	✓	✓	✓	✓

^a^optical microscopy allows visualization of metals soap aggregates and fatty acid efflorescence, and characterization of some pigments ^b^GCMS Meth Prep (II) analysis involves hydrolysis of ester bonds and metal carboxylates, followed by methylation. Therefore, non-polymerised fatty and di-acids present as part of a glyceride, as well as free metal carboxylates and free fatty and di-acids will be detected. Reaction yields are not known.

## Results and Discussion

### OM and SEM-EDX analyses of the surface

**Figure 1:**
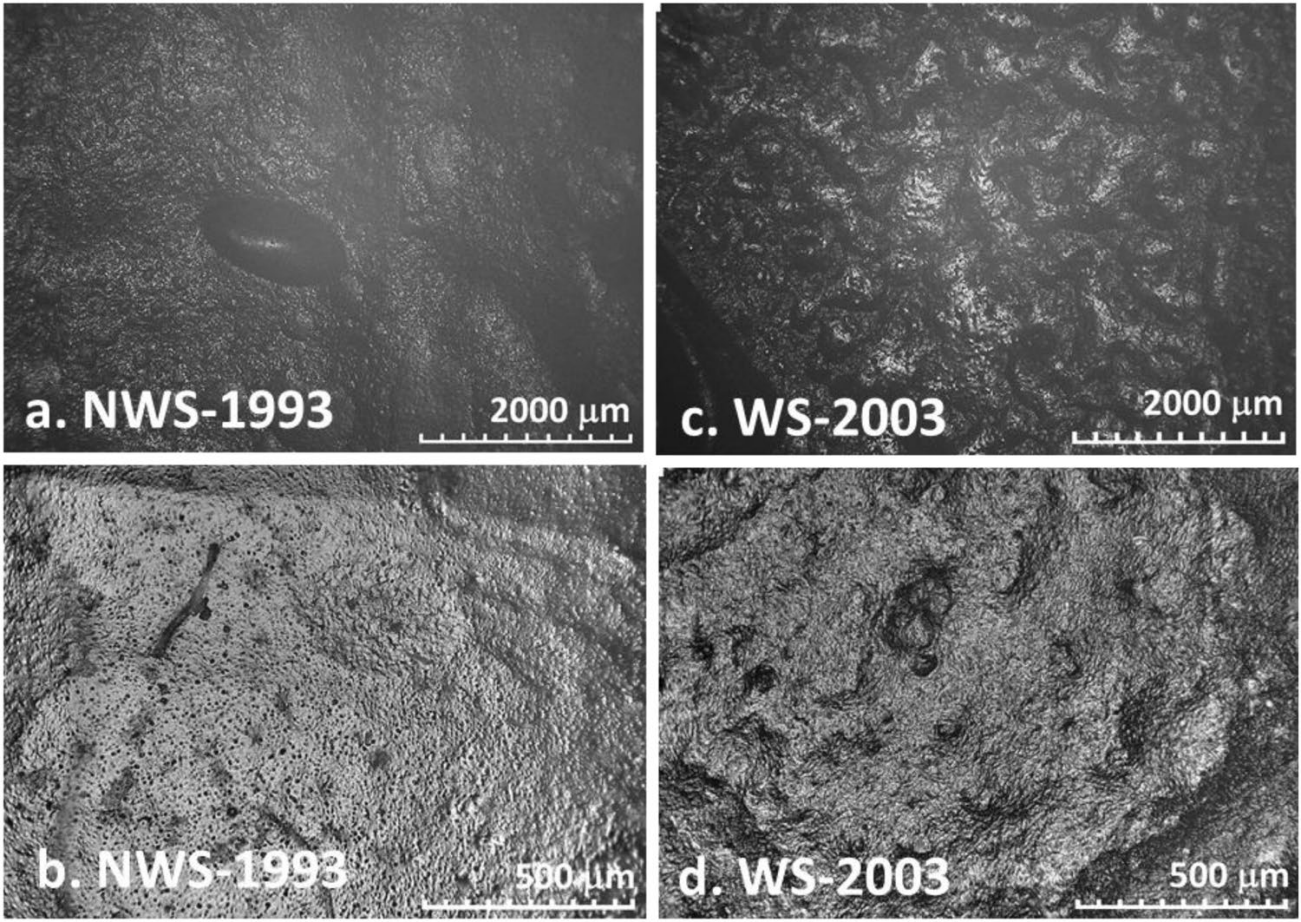
HIROX digital microscope images of NWS-1993 (**a**,**b**; images of the surface at 50x and 200x magnification respectively) and WS-2003 (**c,d**; images of the surface at 50x and 200x magnification respectively).

Both Winsor Green samples (NWS-1993 and WS-2003) appear well-bound and medium-rich when examined with the naked eye. As can be seen in Fig. 1, under high magnification the surface of NWS-1993 has a more uniform and granular appearance than WS-2003, which appears to have a more medium-richer, slightly wrinkled surface.

**Figure 2:**
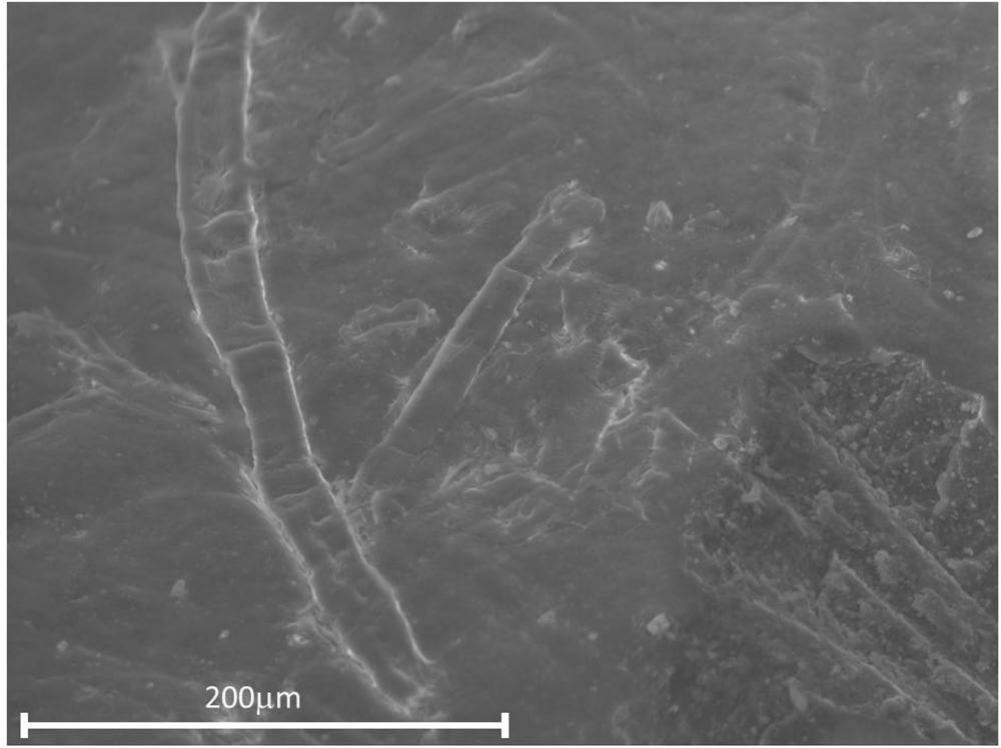
Secondary electron SEM image of Winsor Green 1993.

Although rod-shaped entities^1^ were visible on the surface of the non-water sensitive NWS-1993 sample using electron microscopy (Fig. 2), SEM-EDX elemental mapping (see Supplementary Information, Fig. S1) of both NWS-1993 and WS-2003 revealed fairly homogenous, medium-rich surfaces, with no indications for the formation of epsomite.

The elemental composition of both samples was determined using SEM-EDX (see Table 2). The presence of chlorine and copper is consistent with the phthalocyanine green (PG7) pigment present in both samples (also confirmed using FTIR). Magnesium, barium and sulphur indicated the presence of magnesium carbonate and barium sulphate extenders (as discussed below, both hydromagnesite and barium sulphate were detected using FTIR). Calcium was also identified in both samples indicating possible traces of calcium carbonate, which may suggest a natural source for the hydromagnesite since various calcium and/or magnesium containing carbonate minerals e.g. dolomite, calcite, nesquehonite, and monohydrocalcite are known to form simultaneously. Minor differences were noted between the samples; WS-2003 contained trace levels of chromium (possibly indicating chromium oxide green, Cr_2_O_3,_ pigment) and silica that were not detected in the NWS-1993 sample. The presence of aluminium in both samples may be ascribed to alumina hydrate (hydrated aluminium oxide), known to be included in W&N formulations for Winsor Green artists’ oil colour paints dating to 1965 at levels of ~3% w/w. (accessed via the W&N archive at the Hamilton Kerr Institute, Cambridge, UK). If the formulation has not changed significantly since 1965, the mixture will result in less than 5% w/w. alumina, which would be detectable using EDX, but not necessarily via bulk analysis using FTIR; which is consistent with our results.

**Table 2:** Elemental composition of the Winsor Green swatches.

Sample	SEM-EDX major (trace) elements
NWS-1993	C O Cl Mg Al Ba S (Ca Cu Br)
WS-2003	C O Cl Mg Al Ba S (Ca Cu Br Cr Si)

**Figure 3:**
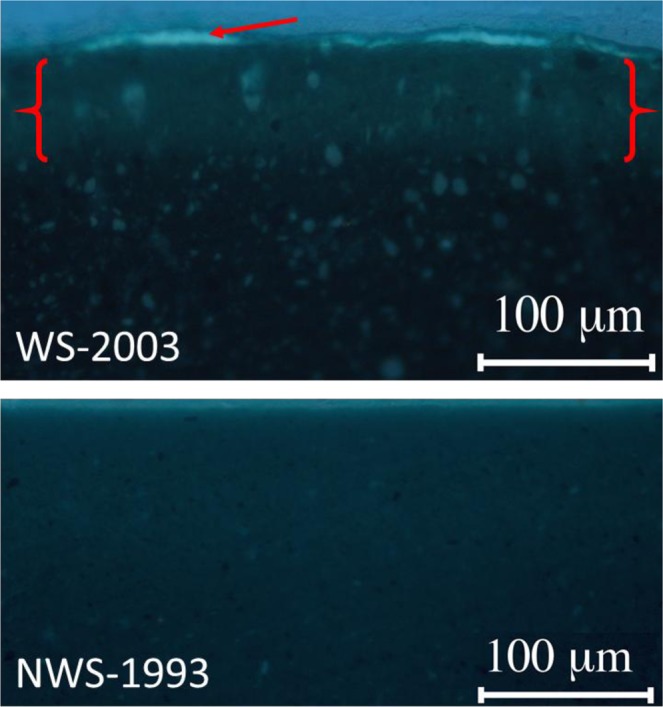
Cross sections of NWS-1993 and WS-2003 shown under UV light (illumination via an Osram HBO 50 W short arc mercury lamp). The 2003 sample has a fluorescent layer (in the region enclosed by the red brackets) extending ~30 µm below the paint surface. The white region indicated by the red arrow is residual embedding resin particulates in the uppermost surface of the sample. Smaller evenly distributed fluorescent inclusions are also visible throughout the bulk of the cross section of WS-2003. As can be seen, these features are absent in NWS-1993 sample.

UV microscopy of embedded paint cross sections identified a distinct ultraviolet (UV) fluorescent layer just below the surface of WS-2003, which was absent in NWS-1993 sample (Fig. 3). This fluorescent layer did not correspond to any features visible in the cross section when viewed under visible light.

SEM imaging and elemental mapping of a cross section of the NWS-1993 sample showed a homogenous distribution of elements. Conversely, the region associated with the UV-fluorescence band in WS-2003 (Fig. 3) included an enrichment of carbon and a reduction of chlorine, sulphur and barium (see Supplementary Information, Figs S1, S2). This indicates a reduction in the concentration of both the phthalocyanine green pigment and barium sulphate extender, with a concomitant enrichment of binding medium in this region, producing a more medium-rich sub-surface.

### FT-IR analyses

**Figure 4:**
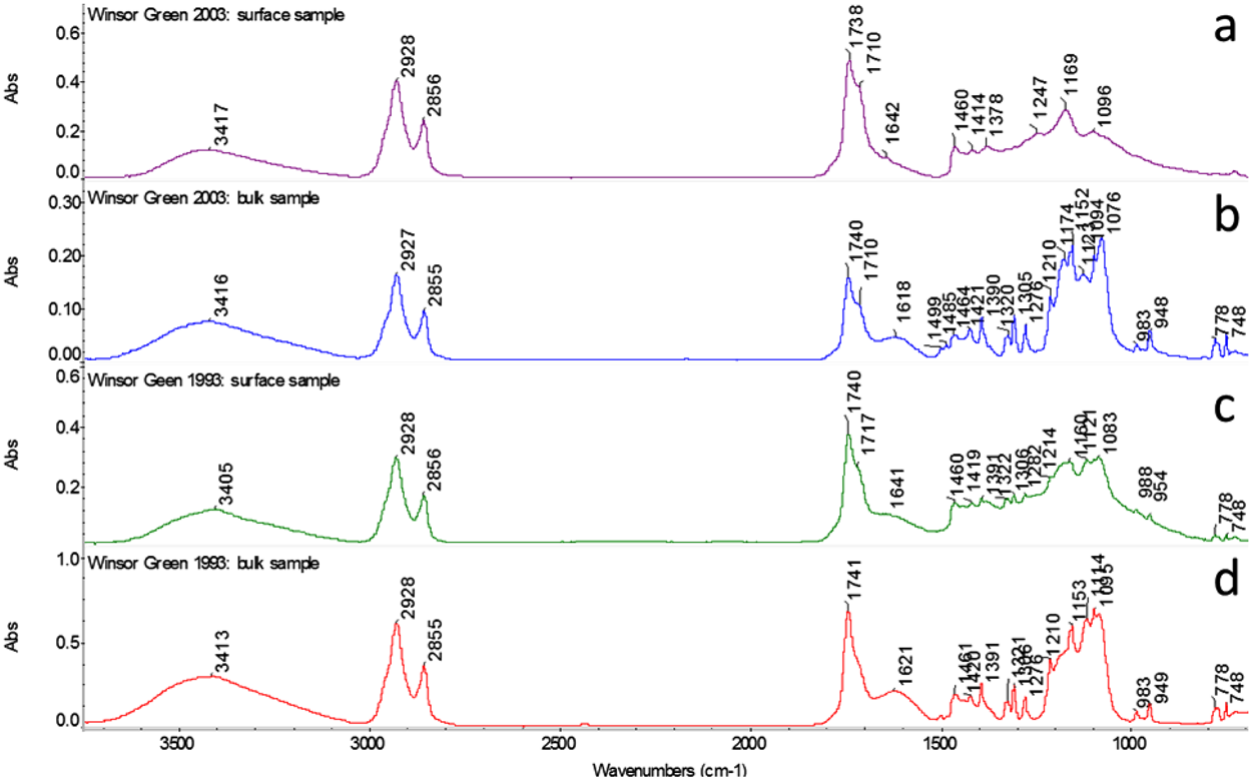
FTIR spectra of (**a**) WS-2003 surface sample (**b**) WS-2003 bulk sample (**c**) NWS-1993 surface sample and (**d**) NWS-1993 bulk sample. Six spectra were acquired for each sample, representative spectra are shown.

FTIR analysis of the bulk (whole film) samples showed that the Winsor Green swatches contained a cured drying oil medium, with phthalocyanine green (PG7), barium sulphate, and low levels of magnesium carbonate (Fig. 4 and Table 3). A broad IR absorption band of low intensity centred at ~1620 cm^−1^ and a sharp band at ~1320 cm^−1^ were present in both NWS-1993 and WS-2003 (see Fig. 4). These absorptions may possibly be ascribed to the formation of metal, and possibly calcium, oxalates, which indicate oxidative degradation of the binder, however adsorbed water (bending vibration) might also contribute to the band at ~1620 cm^−1^. In general, it is noted that the amorphous metal soap content^23^ of both paints is minimal. This is unsurprising given that these paints are made using PG7, which is not known to form metal soaps *in-situ*. However, extender pigments such as calcium carbonate may form metal soaps if exposed to a slightly low pH, and although there is potential for the formation of magnesium and barium soaps in these hydromagnesite and barium sulphate-containing paints, none were found in this study.

**Table 3:** Summary of band assignments for the FTIR spectra shown in Fig. 4 (above).

Absorption bands (cm^−1^)	Assignment
3413 (−OH stretch); 2927, 2855 (CH stretch); 1740 (ester carbonyl absorption); 1711 (C-O stretch carboxylic acids); 1464 (CH_3_-O)	Oil
1499, 1390, 1320, 1390, 1276, 1210, 1094, 948, 778, 748	Phthalocyanine Green (PG7)
1174, 1123, 1076, 983	Barium sulphate
3648 [Mg(OH)_2_; O-H vibration], 1485, 1421 [CO_3_^2−^/HCO_3_^−^; v_3_ asymmetric stretching vibration]	Hydromagnesite Mg_5_(CO_3_)_4_(OH)_2_
1618, 1320	Possible metal [Ca?] oxalate

Finally, the carbonyl absorption of WS-2003 shows a relatively pronounced shoulder at ~1710 cm^−1^ in comparison to NWS-1993 (Fig. 4). This suggests that the water-sensitive sample is relatively rich in free acidic moieties compared with the non-water-sensitive sample, which may be due to a higher extent of hydrolysis, a higher content of dicarboxylic acids (from oxidation), or both. Comparison of the FTIR spectra of surface and bulk samples (Fig. 4) provides further evidence for a more medium-rich surface in WS-2003 (SEM images). Comparison of Fig. 4c with Fig. 4d shows less difference between the surface and bulk FTIR spectra of NWS-1992. However for WS-2003, the surface spectrum only (Fig. 4a) shows absorptions for the ester triplet of oils (at 1247, 1169 and 1096 cm^−1^), as well as C-H bands at 1460 cm^−1^ and 1378 cm^−1^. Absorptions assigned to the PG7 pigment (see Table 3), barium sulphate and magnesium carbonate are only visible in the spectrum of the bulk of WS-2003 and not present in the spectrum acquired at the surface. Combined, the light and electron microscopy images and FTIR data indicate that the NWS-1993 paint is fairly homogeneous, and that the WS-2003 paint is less so.

### Binding medium analysis of the soluble fraction

The chromatographic profiles of both samples (Fig. S.5 Supplementary Information) were characterized by the presence of the following triglycerides: PPO, OOP, OLS, PPS, OSP, OOO, PSS, OOS, OSS, SSS, as well as ArSO and ArOO as the main un-oxidized triglycerides (Acyl substituent abbreviations: C n° of carbon atoms: n° of unsaturation, n° of OH; Ar: Arachidyl (C_20_); L: linoleyl (C_18:2_); O: oleyl (C_18:1_); S: stearyl (C_18:0_); P: palmityl (C_16:0_)). The lipid profiles were also characterized by the presence of oxidized diglycerides (OxDAGs) and triglycerides (OxTAGs) and the main species are reported in Table 4. Based on the TAGs detected, the source of the oils used in both paints were identified as a mixture of linseed and safflower. In addition, the WS-2003 paint was characterized by the presence of specific markers for castor wax, C_18 (OH_C_18 (OH)_C_18(OH)_ (m/z 961.7, [M + Na]^+^) and C_18 (OH)_C_18(OH)_S (m/z 945.7, [M + Na]^+^). Castor wax is often used in modern oil paint formulations as a rheology modifier and has been previously identified in both non-water-sensitive and water-sensitive paints^7^.

**Table 4:** Main oxidized diglycerides (OxDAGs) and triglycerides (OxTAGs) identified in the samples.

Class	Formula
*OxDAGs*	OC_18:1(OH)_
	SC_18:1(OH)_
	SC_18(OH)_
	SC_18:1(2OH)_
	SC_18(2OH)_
*OxTAGs*	POC_18:2(OH)_
	POC_18:1(OH_
	PC_18:2(OH)_C_18:2(OH)_
	PC_18:1(OH)_C_18:2(OH)_
	PC_18:1(OH)_C_18:1(OH)_
	PC_18:1(OH)_C_18(OH)_
	PC_18 (OH)_C_18(OH)_
	OOC_18:2(OH)_
	OOC_18:1(OH)_
	OOC_18(OH)_

Identification was performed via mass spectra interpretation and current literature^21,33–35^.

The content of lauric, myristic, palmitic, oleic and stearic acids were determined via a quantitative HPLC-MS method described in a previous publication, where the absolute amounts determined were normalised for the total mass of the sample. Results indicate that free fatty acids constitute 0.4% by weight of NWS-1993, and 0.8% by weight of WS-2003.

The samples were then analysed by a GC-MS derivatisation approach based on the use of HMDS for the quantitation of free mono- and di-carboxylic acids and BSTFA for the quantitation of the free mono and di-carboxylic acids, plus the unbound metal carboxylates of mono and di-carboxylic acids. Figure S.6 (Supplementary Information) includes the related chromatograms. Quantitation was performed by means of calibration curves and the data are compared with equivalents obtained via HPLC-MS in Table 5, which also includes the methylated GC-MS results obtained from paint fragments, after hydrolysis and methylation, using Meth-Prep II.

**Table 5:** Ratios of fatty and dicarboxylic acids derived from GC-MS and HPLC-MS analysis, and their % content with respect to the mass of the sample.

	Fraction elucidated	GC-MS				HPLC-MS	
		A/P	P/S	FA + DiAc %	FA%	P/S	FA %
1993	FFAs	1.5	1.4	0.7	0.4	1.3	0.4
	FMS + FFAs	1.5	1.5	0.8	—	—	—
	Whole sample (after hydrolysis)	1.4	1.8	0.9	—	—	—
2003	FFAs	0.8	1.3	1.6	0.9	1.4	0.8
	FMS + FFAs	0.9	1.5	1.6	—	—	—
	Whole sample (after hydrolysis)	1.0	1.7	1.6	—	—	—

(FFA = free fatty and dicarboxylic acids, FMS = free metal soaps of fatty and dicarboxylic acids). The fatty acids relative abundances are reported in Table S1 in the supplementary materials.

The combined results indicate that there is no significant proportion of free metal soaps of fatty and dicarboxylic acids in these paints and the dicarboxylic and fatty acid profile of the unbound (unpolymerized) fraction is very similar to that of the whole sample after hydrolysis (see Table 5). This indicates that the di- and mono-carboxylic acids have undergone similar hydrolysis rates, and that the paint formulations did not include significant amounts of free fatty acids. Although the WS-2003 contains 0.4% more free and dicarboxylic acids (combined content of lauric, myristic, palmitic, stearic, suberic, sebacic and azelaic acids) than the NWS-1993; this difference is unlikely to account for the distinct difference in water-sensitivity between these two paints.

This was also the case for the dicarboxylic acids (combined content of suberic, sebacic and azelaic acids). The NWS-1993 sample contained ~0.3% w/w. free dicarboxylic acids (mainly azelaic acid, as shown by the chromatographic profiles in Fig. S.6 supplementary) with respect to the sample weight, while WS-2003 contained about 0.7%. This minimal difference is unlikely to account for the marked differences in water sensitivity, as, although the content of free dicarboxylic acids in WS-2003 is double than that NWS 1993, they are both below 1% of the sample weight^6^. Assuming a paint sample containing 1 g of paint binder, WS-2003 contained 7 mg of the slightly water soluble azelaic acid, and 993 mg of glycerides and insoluble matter; whereas NWS-1993 contained 3 mg of azelaic acid, and 997 mg of glycerides and insoluble matter. This suggests that even if water sensitive oil-paints are vulnerable to the extraction of dicarboxylic acids when exposed to water and protic solvents, water-sensitivity is not necessarily caused primarily by the absolute content of the unbound dicarboxylic acids in the paint, particularly given that the polarity of the paint network appears to be largely determined by the polarity of the crosslinked material, as is discussed below. However, it is also acknowledged that the exact mechanism(s) of water-transport and diffusion into water-sensitive (polar) paint films requires further investigation.

Given that there appears to be no significant difference in the metal soap content of the two Winsor Green paint samples, and that the content in dicarboxylic acids is below 1% in both samples, the cause of water sensitivity of WS-2003 must be largely unrelated to the presence/absence of metal soaps, and the composition of the free mono and dicarboxylic acids. As discussed earlier, FTIR analysis indicated that WS-2003 presents a higher content of acidic moieties than NWS-1993. Based on the quantitative results obtained via HPLC-MS and GC-MS, the data suggests that the free acidic moieties must originate from fatty acids covalently bound to the polymeric network and are thus not extractable or hydrolysable. Since polyunsaturated fatty acids contain more than one double bond, they are capable of undergoing cross-linking reactions and oxidative scission at different double bond locations. Oxidised polyunsaturated fatty acids can therefore be bound to the crosslinked polymer at one location, and have a free carboxylic acid functional group at another location.

### Binding medium analysis including the polymeric network

**Figure 5:**
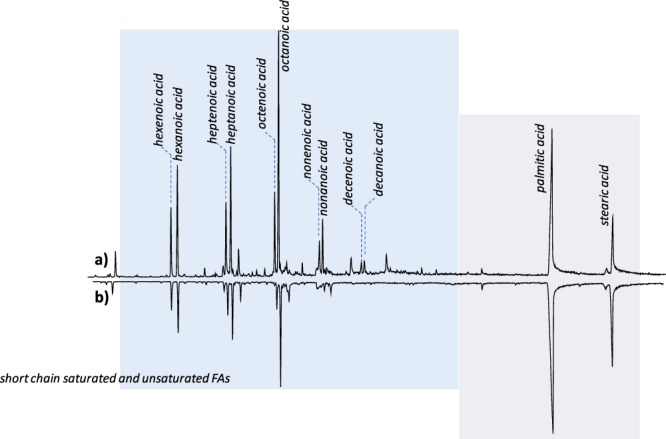
Py-GC-MS chromatograms obtained for the two Winsor Green paint layers: (**a**) NWS-1993 (**b**) WS-2003.

To investigate the polymeric network further at the molecular level, analytical pyrolysis was used. Figure 5 shows the extracted ion pyrograms of the fragment ion at *m/z* 129, which is known to be abundant in the mass spectra of TMS-esters of fatty acids (data has been normalised to the intensity of the palmitic acid peak).

The pyrograms in Fig. 5 can be divided in two different regions, one characterised by the presence of short-chain saturated and unsaturated fatty acids (C4-C10), deriving from the pyrolysis of the polymeric network, and the second characterised by the presence of palmitic and stearic acids, which are unsaturated and thus not subject to auto-oxidative phenomena. Although it is known that pyrolytic profiles and derivatisation yields with HMDS are strongly influenced by the whole sample composition (including the inorganic content)^24,25^, given the high similarity of the two samples, it was possible to carry out a semi-quantitative comparison of the two pyrograms.

WS-2003 was characterised by relatively lower amounts of pyrolysis products deriving from the polymeric network, when compared to NWS-1993. This points to a difference in the extent of the polymeric network between the two paints and suggests that NWS-1993 is more polymerised.

Evolved gas analysis was also performed as a complementary analytical approach, where the gases evolved from a progressively heated sample undergoing decomposition or desorption are characterised^26,27^. Evolved gases formed during heating are directly transferred into the mass spectrometer by a deactivated open transfer line, providing information on the molecular nature of the fractions with different thermal stability.

**Figure 6:**
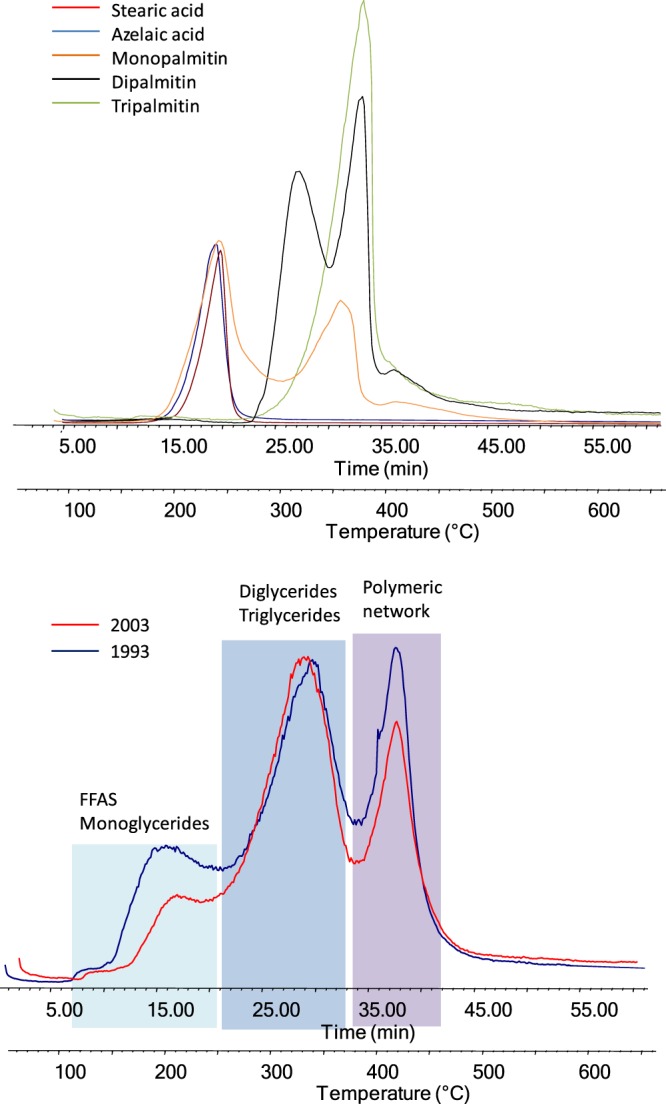
EGA-MS profiles obtained for a set of standards (stearic acid, azelaic acid, monopalmitin, dipalmitin and tripalmitin) and EGA-MS profiles obtained for the two Winsor Green paint layers NWS-1993 (blue), WS-2003 (red). The mass spectra of the thermal zones are reported in the supplementary information (Figs S8–S13).

In the operating conditions used (m/z 50–700) EGA-MS is only sensitive to the thermal degradation of organic materials, whilst the thermal decomposition of the inorganic materials present – leading for example to decarboxylation - does not affect the total ion thermogram. The phthalocyanine green (PG7) pigment is thermally stable and degradation commences only after 550 °C, as shown in the TGA curve (Supplementary Information Fig. S.7). At 600 °C, PG7 shows a mass loss of about 10% where the thermal decomposition is dominated by the loss of hydrochloric acid (monoisotopic mass 36 Da), and minor amounts of aromatic compounds^28^. As a result, the EGA-MS curves at up to 550 °C relate only to thermal decomposition of different fractions of the oil binder. The EGA-MS curves shown in Fig. 6 have been compared to reference standards (stearic and azelaic acid, monopalmitin, dipalmitin and tripalmitin).

Based on the profiles of reference standard samples (Fig. 6), the EGA profiles can be divided in three different thermal zones:

- below 250 °C:fatty and dicarboxylic acids are desorbed, and monoglycerides are partially degraded. 3
- 250 °C - 400 °C: thermal degradation of acylglycerides occurs and
- above 400: average mass spectrum characterised by fragment ions at *m/z* 91 and 105, ascribed to alkylated benzenes.

These mass spectral features, together with the temperature at which thermal decomposition occurs, indicate that the third thermal degradation step (above 400 °C) is due to the decomposition of the cross-linked network. Figure 6 clearly shows that NWS-1993 has a relatively higher cross-linked content, and a relatively lower content of acyl glycerides; concurring with the interpretation of the Py-GC-MS profiles.

## Conclusions

To conclude, the samples studied in this work contained as a main pigment PG7, an organic pigment, and presented very similar inorganic composition (as determined by SEM-EDX and FTIR analysis), but for trace elements, which might have contributed to the curing of the oil, but not to the establishment of an extensive ionomer-like network.

The combined microscopy and analytical investigations of these two very similar paint samples, but with marked differences in water sensitivity, has produced new information that has helped our understanding of the factors that can influence water sensitivity in oil paints.

As epsomite was not detected at the surface of either sample, this is not a primary cause of water-sensitivity in this case. Similarly, water sensitivity does not appear to relate to a higher abundance of hydrolysed free fatty acids or dicarboxylic acids in the oil fraction, nor the formation of an ionomer-like network, as metal soaps were not detected. Sample WS-2003 also contained castor wax. Although it cannot be excluded that castor wax may affect the curing of the oil, the detection of castor wax in both water sensitive and non-water sensitive oil paints in other studies does not suggest a strong correlation with water sensitivity^7^.

Water-sensitivity appears to be primarily related to the degree of polymerisation (i.e. covalently crosslinked network) of the oil and to the polarity of the polymerised fraction (i.e. presence of hydroxyl and di - acids covalently bound to the polymeric network). The non-water sensitive sample (NWS 1993) was more cross-linked and consisted of a relatively non-polar polymeric network; whereas the water sensitive sample (WS 2003) was less cross-linked and the corresponding polymeric network was more polar. This may help to explain the observation that the water-sensitive paint (WS 2003) had a more medium-rich surface in comparison to the non-water-sensitive paint (NWS 1993), possibly through the migration and/or syneresis of organic material toward the paint surface in the WS-2003 sample^29,30^. In addition, given the differences in the degree of polymerisation between the NWS-1993 and WS-2003 paint samples, it is reasonable to propose that water may penetrate and diffuse more easily into the less cross-linked and more polar paint film, disrupting structural integrity, and facilitating the extraction of soluble and/or polar compounds and disruption to paint surfaces, which will be the subject of further research.

## Materials and Methods

### Chemicals

Hexadecane, tridecanoic acid (purity 99%), (N,O-bis(trimethylsilyl)trifluoroacetamide (BSTFA) containing 1% trimethylchlorosilane (TMCS) and 1,1,1,3,3,3-hexamethyldisilazane (HMDS) were all purchased from Sigma-Aldrich (U.S.A.). For the GC-MS quantitative analysis solution was prepared in acetone containing lauric (4.10 µg/g), suberic (4.27 µg/g), azelaic (3.95 µg/g), myristic (4.11 µg/g), sebacic (3.85 µg/g), palmitic acid (4.39 µg/g), oleic acid (6.32 µg/g) and stearic (6.62 µg/g) acids. All standard solutions were used to derive calibration curves. The acids were purchased from Sigma-Aldrich, purity >99%. A solution of tridecanoic acid (purity 99%; Sigma-Aldrich) in iso-octane, 139.91 µg/g, was used as internal standard for derivatization; a solution of hexadecane (purity 99%; Sigma-Aldrich) in iso-octane, 142.00 µg/g, was used as internal standard for injection.

The solvents used as eluents were iso-propanol, water and methanol (HPLC-MS grade; Fluka). For the HPLC-MS quantitative analysis, a stock fatty acid solution was prepared in acetone (HPLC grade; Sigma-Aldrich), and stored at 4 °C in the dark. It contained lauric acid (0.21 µg/g), myristic acid (0.16 µg/g), palmitic acid (0.19 µg/g), oleic acid (0.26 µg/g), linoleic acid (0.20 µg/g), linolenic acid (0.18 µg/g) and stearic acid (0.26 µg/g). This solution was used to derive the calibration curves in the concentration range 3–400 ng/g. The acids were purchased from Sigma-Aldrich (purity >99%). For derivatization, 2-hydrazinoquinoline (HQ), triphenylphosphine (TPP, purity 99%), and 2,2′-dipyridyl disulfide (DPDS, purity 98%) were purchased from Sigma-Aldrich (USA); solutions of 70.20 µg/g HQ, 69.00 µg/g DPDS and 79.50 µg/g TPP were prepared in acetonitrile (LC-MS grade, Sigma Aldrich) and stored at 4 °C in the dark. Monopalmitin, dipalmitin, and tripalmitin used for the EGA-MS analysis were purchased from Sigma-Aldrich (purity >99%).

### Paint samples

Two Winsor & Newton (W&N) Winsor Green [phthalocyanine green (PG7)] Artists’ Oil Colour paint swatches dating to 1993 and 2003 were selected for this study. Both swatches were painted onto unprimed paper supports using a draw-down bar to create both a thick (~ 0.5 mm) and thinner layer of paint, with the samples analysed taken from the thicker region. Pigment weight % content for the paint from 2003 is 28%, and for the paint from 1993 is 29%. They belong to a group of 27 naturally aged W&N Artists’ Oil Colour swatches dating from 1945–2003 which were previously investigated to understand the development of water sensitivity^7^. The swatches were produced at the former Winsor & Newton factory (Greater London, UK) to monitor the drying behaviour of formulations prior to upscaling to larger batches. These ‘swatches’ were then pinned to the wall of the quality control laboratory and allowed to dry (while exposed to ambient light and temperature conditions) before being transferred to dark storage in uncontrolled conditions on factory premises. The swatches were later donated to Tate, where they form part of the wider Winsor and Newton archive. The environmental exposure history of the two samples used in this study is therefore unknown and is likely to have influenced the curing behaviour and ageing of the two paints investigated.

### Cross sections preparation and optical microscopy

Samples were embedded in a polyester resin (Clear Casting AM resin; Tiranti LTD UK.) and cured using a liquid hardener (Butanox M-50; methyl ethyl ketone peroxide solution in dimethyl phthalate; Tiranti LTD, UK). Embedded samples were then ground using abrasive paper (silicon carbide paper) in order to reveal a cross sectional surface, which was then further smoothed using Micromesh**™** polishing cloths^31^. Cross sections were photographed in visible light (illumination provided by an OSRAM Xenophot® 12 V 100 W halogen bulb, with a colour temperature of 3300 K) and UV light (illumination provided by a Osram HBO® mercury vapour short arc 50 W bulb) using a Leica DMR-X microscope.

### Environmental Scanning Electron Microscopy with Energy-Dispersive X-ray Spectroscopy (ESEM-EDX)

ESEM-EDX was carried out using a Philips XL30 ESED-FEG instrument fitted with an Oxford INCA EDS analysis system. The EDS analysis was done under standard ESEM conditions: H20 mode, 10 mm, 20 kV accelerating voltage, 0.7 torr water vapour pressure.

### Fourier transform infrared spectroscopy (FTIR)

Transmission FTIR analysis was carried out on a Thermo scientific Nicolet iN10 MX microscope with a single diamond cell, equipped with a MCT-A/CdTe detector. 64 scans were collected at a resolution of 4 cm^−1^ across a wavenumber range of 4000 to 675 cm^−1^. The collection time was 22 s. Data was obtained and processed using OMNIC 9 software.

### HPLC instrument conditions and sample pre-treatment (HPLC-MS)

For the HPLC analysis, ~0.1 mg of each sample was subjected to extraction assisted by microwaves in a microwave oven Ethos One (Milestone, U.S.A.) (power 600 W), with 300 µL of a chloroform-hexane (3:2) mixture at 80 °C for 25 min.

Carboxylic acids were derivatised using 2,2′-dipyridyl disulphide (DPDS) and triphenylphosphine (TPP), and 2-hydrazinoquinoline (HQ) to form hydrazides. For the derivatisation reaction, 20 μL of the extraction solutions were dried under nitrogen flow in the insert of a 2 mL auto-sampler vial, dissolved with 60 µL of acetonitrile to which was added 20 µL of HQ, DPDS, and TPP containing solutions. The sample were heated for 60 °C for 6 hours^32^. All the analyses were carried out on a 1200 Infinity HPLC coupled by a Jet Stream ESI interface with a Quadrupole-Time of Flight tandem mass spectrometer 6530 Infinity Q-ToF detector (Agilent Technologies, USA). The separation and mass spectrometric experiments were performed according to the conditions reported in^21,33–36^. The fatty acid quantification was based on calibration curves reporting the integrated areas of the [M + H]^+^ ions of the HQ derivatives as detected by the “Find by Formula” algorithm. The interpretation of the acyl glyceride species was performed by comparison with the literature^21,33–37^.

### Gas Chromatography-Mass spectrometry (GC-MS) analysis of metal carboxylates and free fatty acids

For the analysis of free fatty acids, 5 µL of tridecanoic acid solution was added to the samples, which were then dried under nitrogen flow at room temperature in order to remove the solvent. The residual solid was derivatised using 20 µL of HMDS, 150 µL of *iso*-octane at 60 °C for 30 min. 5 µL of hexadecane solution was added just before injection, as an injection internal standard. For the analysis of metal carboxylates, the mixture obtained following the derivatisation with HMDS was dried under nitrogen flow at room temperature, and subsequently 20 µL of BSTFA and 150 µL of *iso*-octane was added. The reaction time and temperature were set at 81 min and 78 °C^7^. GC-MS instrumentation consisted of an Agilent Technologies 6890 N Gas Chromatograph coupled with a 5973 Mass Selective Detector single-quadrupole mass spectrometer. Samples were injected in splitless mode at 280 °C. GC separation was performed on a fused silica capillary column HP-5MS (J&W Scientific, Agilent Technologies, stationary phase 5% diphenyl-95% dimethyl-polysiloxane, 30 m length, 0.25 mm i.d., 0.25 μm film thickness). The chromatographic were the same to those reported in^7^.

### GC-MS analysis of total fatty acid content (excluding the crosslinked fraction)

Surface scrapings (of less than 1 mg) and bulk samples (of around 1 mg) were taken from the Winsor Green paints. Samples were derivatised using Meth Prep II (Grace™ Alltech™). Details on the analytical procedure are reported elsewhere^38^. GC was carried out on a Varian CP-3900 GC coupled with a 1200 L MS detector. Oven program: 80 °C ramped to 320 °C at 10 °C/min then held for 5 minutes at 320 °C. Total run time was 29 minutes. Split (20:1) injection volume was 1 μl, and the helium flow was 1.0 ml/min. MS conditions: source temperature: 220 °C; transfer line temperature: 270 °C, injection port set at 300 °C. Column: Phenomenex Zebron ZB-5 column (30 m length; 0.25 mm i.d.; 0.25 µm film thickness). EI mode (70 eV); scan group 1: 45–300 amu; Group 2: 45–700 amu at 16 mins, every 1 s. Chromatographic peak areas were used to calculate ratios between the abundances of significant analytes.

### Pyrolysis-Gas Chromatography-Mass Spectrometry (Py-GC-MS)

Analyses were performed using a multi-shot pyrolyzer EGA/PY-3030D (Frontier Lab, Japan) coupled with a 6890 N gas chromatography system with a split/splitless injection port and combined with a 5973 mass selective single quadrupole mass spectrometer (Agilent Technologies, U.S.). The samples were placed in platinum sample cups on glass wool. HMDS was added to the samples and placed on top of the pyrolyzer at room temperature, and then quickly introduced in the pyrolysis chamber. Pyrolysis conditions were as follows: pyrolysis chamber temperature 550 °C, interface 280 °C^39,40^. The GC injector temperature was 280 °C. The GC injection port operated in split mode and the best analytical results were obtained with a split ratio of 1:10. The chromatographic separation of pyrolysis products was performed on a fused silica capillary column HP-5MS (5% diphenyl-95% dimethyl-polysiloxane, 30 m × 0.25 mm i.d., 0.25 μm film thickness, J&W Scientific, Agilent Technologies), preceded by 2 m of deactivated fused silica pre-column with internal diameter of 0.32 mm. The chromatographic conditions for the analysis were: 32 °C for 10 min, 10 °C/min to 280 °C, 300 °C for 2 min, 15 °C/min to 300 °C. The helium (purity 99.9995%) gas flow was set in constant flow mode at 1.2 mL/min. MS parameters: electron impact ionization (EI, 70 eV) in positive mode; ion source temperature 230 °C; scan range 50–700 m/z; interface temperature 280 °C. Perfluorotributylamine (PFTBA) was used for mass spectrometer tuning^41^. MSD ChemStation (Agilent Technologies) software was used for data analysis and peak assignment was based on the comparison with libraries of mass spectra, (NIST 1.7, WILEY275) and mass spectra interpretation.

### Evolved Gas Analysis-Mass Spectrometry (EGA-MS)

Samples, ranging in mass from 100 to 300 µg, were placed into a stainless-steel cup and inserted into the microfurnace. The instrumentation consisted of a micro-furnace Multi-Shot Pyrolyzer EGA/Py-3030D (Frontier Lab, Japan) coupled with a gas chromatograph oven 6890 Agilent Technologies (Palo Alto, USA) equipped with a deactivated and uncoated stainless-steel transfer tube (UADTM-2.5 N, 0.15 mm i.d. × 2.5 m length, Frontier Lab). The transfer tube was coupled with a 5973 Agilent Mass Selective Detector single quadrupole mass. Temperature program for the micro-furnace chamber: initial temperature 50 °C; 10 °C/min up to 800 °C. Analyses were performed under a helium flow (1 ml/min) with a split ratio 1:20. The micro-furnace interface temperature was automatically kept at 100 °C higher than the furnace temperature until the maximum value of 300 °C. The inlet temperature was 280 °C. The chromatographic oven was kept at 300 °C. The mass spectrometer was operated in EI positive mode (70 eV, scanning m/z 50–700). The MS transfer line temperature was 300 °C. The MS ion source temperature was kept at 230 °C and the MS quadrupole temperature at 150 °C.

## Supplementary information

Supplementary information
