# Supplementary material for: The role of the polymeric network in the water sensitivity of modern oil paints
Source: Sci Rep. 2019 Mar 5;9:3467. doi: 10.1038/s41598-019-39963-z (PMC6400961; doi:10.1038/s41598-019-39963-z)
Supplement: Supplementary file 1 — Supplementary information [file 41598_2019_39963_MOESM1_ESM.pdf]

Supplementary Information

The role of the polymeric network in the water sensitivity of modern oil paints

Jacopo La Nasa, Judith Lee, Ilaria Degano, Aviva Burnstock, Klaas Jan van den Berg, Bronwyn Ormsby, Ilaria Bonaduce

Figure S.1 Winsor Green, 2003: cross section sample (water-sensitive)

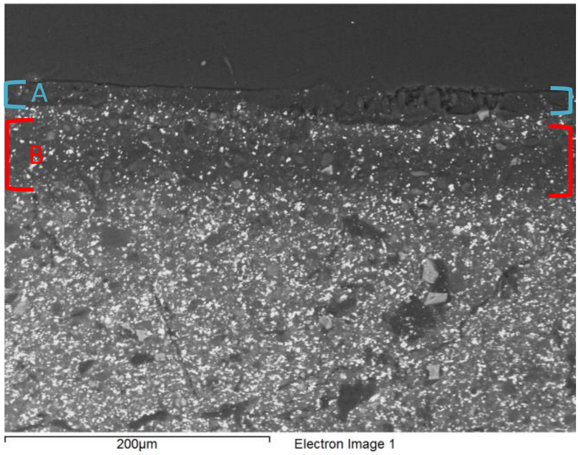

Backscattered SEM image of a cross-section sample of Winsor Green 2003 (above) with corresponding SEM-EDS maps a-k.

The elemental map of carbon (map a) shown to the right indicates an enrichment of carbon containing organic material toward the upper surface in two main regions. These regions are labelled A and B in the SEM image shown above. Region 'A' represents a carbon rich skin of medium at the very surface that is frequently observed in water-sensitive oil paint samples. The sub-surface region 'B' represents a carbon-rich region, with a relatively decreased concentration (relative to bulk areas of the sample) of Al (map d), S (map f), Cl (map g), and Ba (map k). These elements are believed to correspond to alumina (aluminium oxide), pthalocyanine green pigment (which contains chlorine), and barium sulphate. This suggests the upper surface is more rich in organic material, and deficient in solids content. UV-light microscopy of the cross section (see Figure 3 in main manuscript) indicates the carbon-enriched areas have a UV fluorescence, unlike the bulk portion. Taken together this indicates phase separation behavior has taken place in the water sensitive 2003 sample.

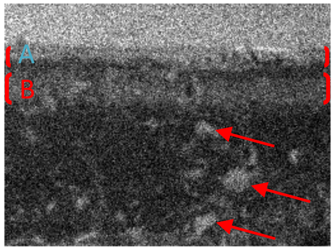

a. SEM-EDS map for C Ka showing enrichment of carbon containing material towards the surface in two 'layers' (indicated by the red brackets, and labelled A and B), and also in discrete areas within the bulk of the sample (examples are indicated with the red arrows).

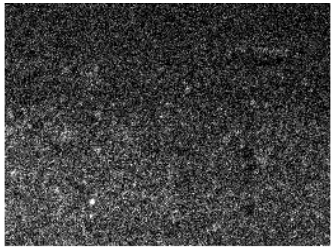

b. SEM-EDS map for carbon, O Ka showing a homogenous distribution.

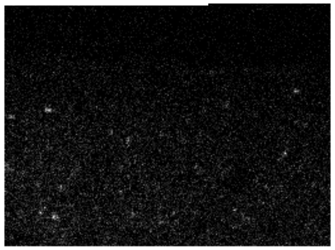

c. SEM-EDS map for carbon, Mg Ka showing a fairly homogenous distribution.

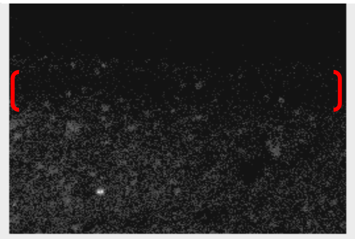

d. SEM-EDS map for Al Ka. Some larger clusters of aluminium-containing particles are visible, and a general deficiency of aluminium within the sub-surface region (the area indicated by the red brackets) that is rich in carbon.

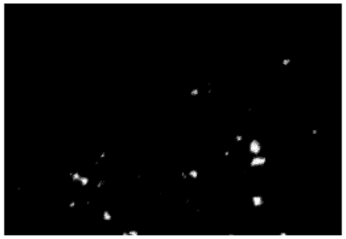

e. SEM-EDS map for Si Ka. Isolated silicon-rich particles (possible silica) are visible within the bulk of the cross section.

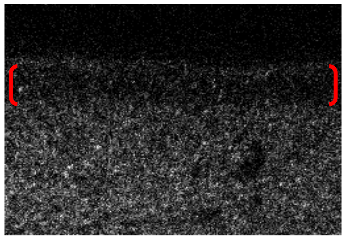

f. SEM-EDS map for S, Ka showing a homogenous distribution except for the sub-surface region (indicated by the red brackets) which is more deficient in sulphur.

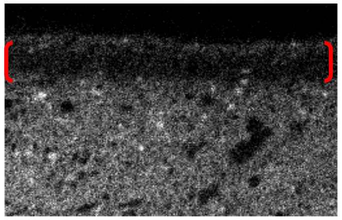

g. SEM-EDS map for Cl Ka . The sub-surface region which is more deficient in Cl (within the area indicated by the red brackets), which is associated with the phthalocyanine green pigment.

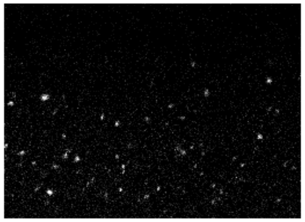

h. SEM-EDS map for Ca, Ka showing a fairly homogenous distribution of calcium with a few larger clusters.

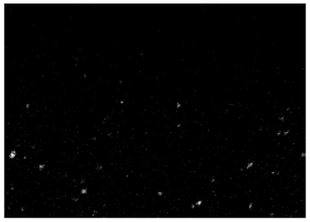

i. SEM-EDS map for Cr Ka. Fairly homogenous, with some small clusters visible.

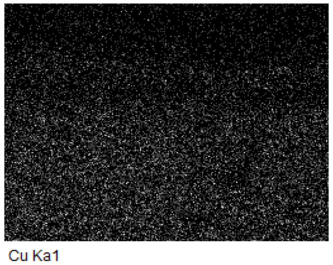

j. (left). SEM-EDS map for Cu, Ka.

k. (right) SEM-EDS map for Ba La. Showing homogenous distribution of barium with the sub-surface area (indicated by the red brackets) more deficient in barium.

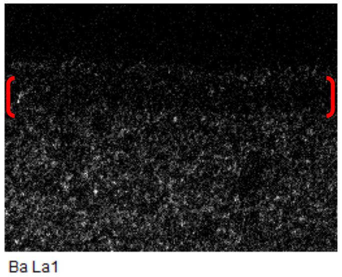

Figure S.2 Winsor Green, 2003: surface sample (water-sensitive)

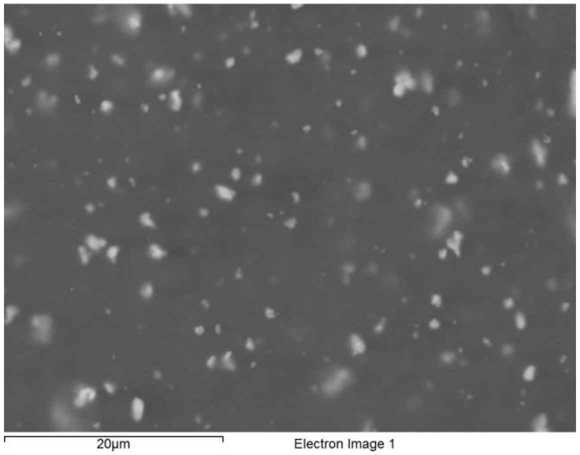

Backscattered SEM image of a surface sample of Winsor Green 2003 (above) with corresponding SEM-EDS maps a-j.

The SEM-EDS elemental maps shown to the right show that the surface of the sample appears quite homogenous. Some regions appear relatively enriched in both barium and sulphur (see in Fig S.4.e and S.4.j) indicating the presence of barium sulphate, that is used as an extender in the paint sample.

There was no indication for the formation of magnesium sulphate salts (epsomite).

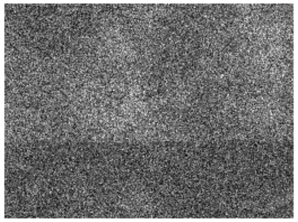

a. SEM-EDS map for C Ka; sample appears homogenous.

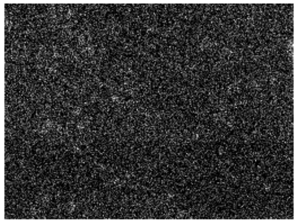

b. SEM-EDS map for O Ka; sample appears homogenous.

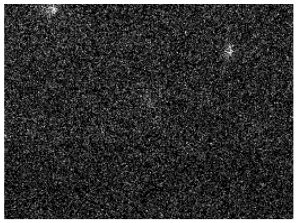

c. SEM-EDS map for Mg Ka; sample appears homogenous.

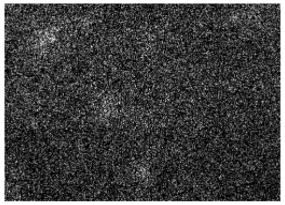

d. SEM-EDS map for Al Ka; sample appears largely homogenous (some minor clustering)

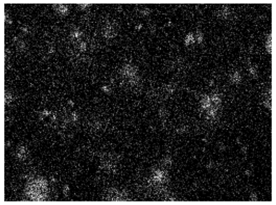

e. SEM-EDS map for S Ka; some sulhur rich regions which correlate to the Ba-rich regions and are likely barium sulphate.

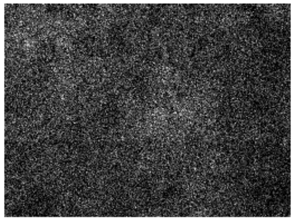

f. SEM-EDS map for Cl Ka; appears homogenous.

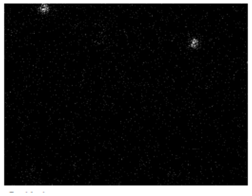

g. SEM-EDS map for Ca Ka; some traces of calcium present.

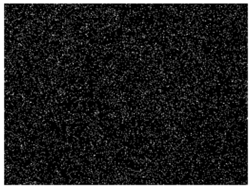

h. SEM-EDS map for Cu, Ka.

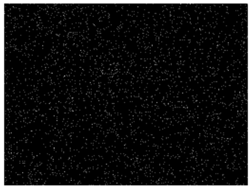

i. SEM-EDS map for Br, Ka.

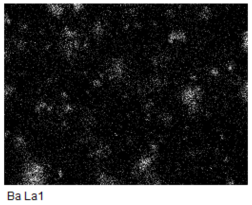

j. SEM-EDS map for Ba La, showing clusters of barium that correspond to areas rich in S.

Figure S.3 Winsor Green, 1993: cross section sample (non-water-sensitive)

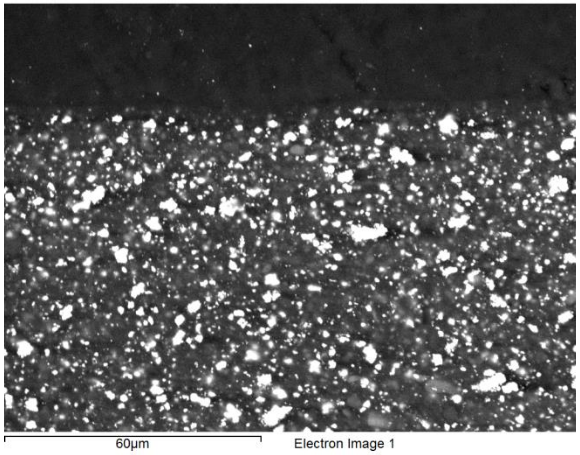

Backscattered SEM image of a cross section sample of Winsor Green 1993 (above) with corresponding SEM-EDS maps a-j.

The SEM-EDS elemental maps for the cross section of Winsor Green 1993 appear far more homogenous than those of the 2003 sample. E.g. the carbon Ka, sulphur Ka, barium La, and chlorine Ka elemental maps all indicated an an even distribution of elements (aside for some clustering associated with agglomerates of barium sulphate extender). Unlike the elemental maps for the 1993 sample, these elemental maps <u>do not</u> indicate a medium rich region present at the sub-surface of the cross section, that is enriched in carbon, and deficient in elements that relate to extender solids and pigment.

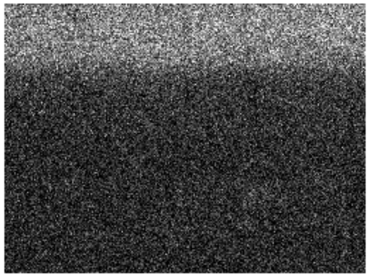

a. SEM-EDS map for C Ka; sample appears homogenous.

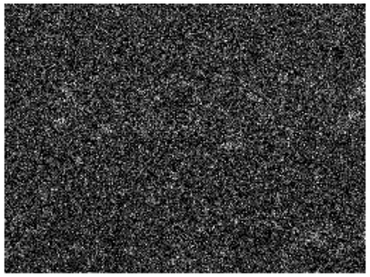

b. SEM-EDS map for O Ka; sample appears homogenous.

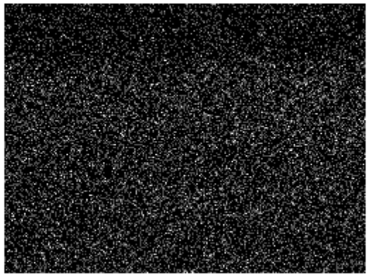

c. SEM-EDS map for Mg Ka; sample appears homogenous.

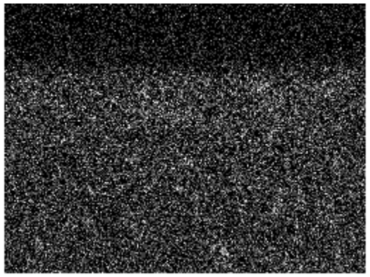

d. SEM-EDS map for Al Ka; sample appears homogenous.

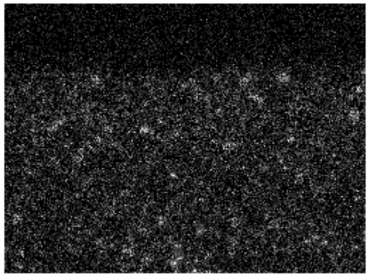

e. SEM-EDS map for S Ka; sample appears fairly homogenous. Some clusters that are more rich in sulphur are visible. These areas also correspond to those rich in barium, indicating barium sulphate.

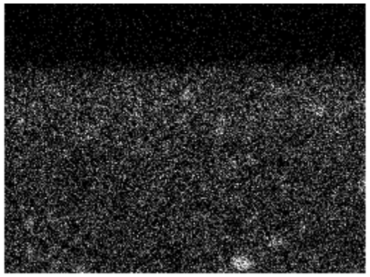

f. SEM-EDS map for Cl Ka; sample appears homogenous.

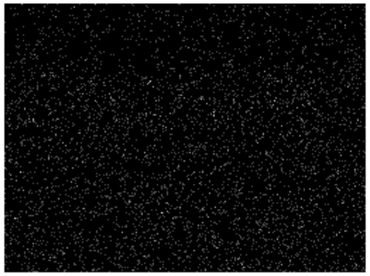

g. SEM-EDS map for Cu Ka; sample appears homogenous.

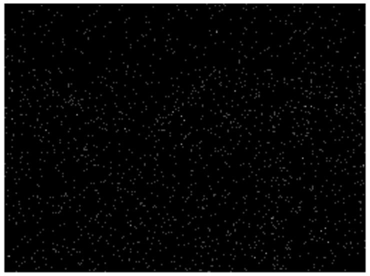

h. SEM-EDS map for Br Ka; sample appears homogenous.

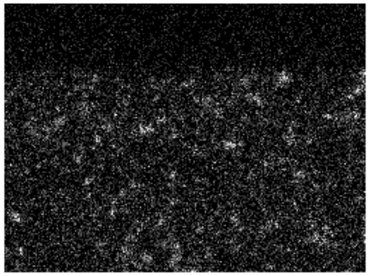

i. SEM-EDS map for Ba La; come clustering of barium-rich entities are visible. These areas correspond to those rich in Sulphur indicating barium sulphate.

Figure S.4 Winsor Green, 1993: surface sample (non-water-sensitive)

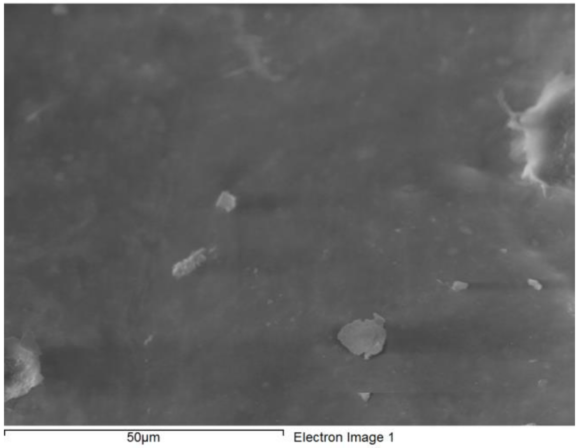

Secondary electron SEM image of a surface sample of Winsor Green 1993 (above) with corresponding SEM-EDS maps a-i.

The SEM-EDS elemental maps for the surface sample of Winsor Green 1993 appear homogenous. There was no indication for the formation of magnesium sulphate salts (epsomite) at the paint surface.

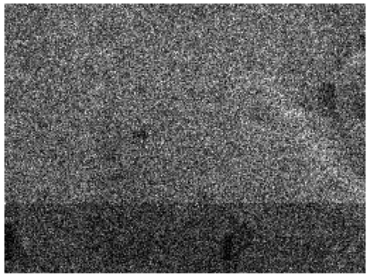

a. SEM-EDS map for C Ka; sample appears homogenous.

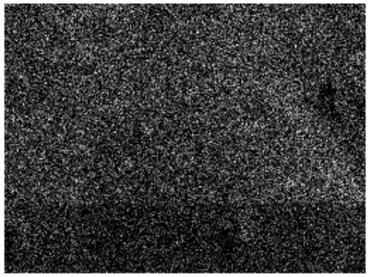

b. SEM-EDS map for O Ka; sample appears homogenous.

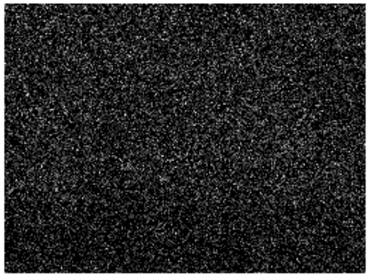

c. SEM-EDS map for Mg Ka; sample appears homogenous.

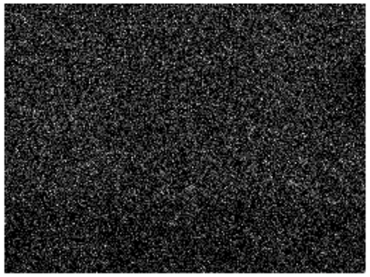

d. SEM-EDS map for Al Ka; sample appears homogenous.

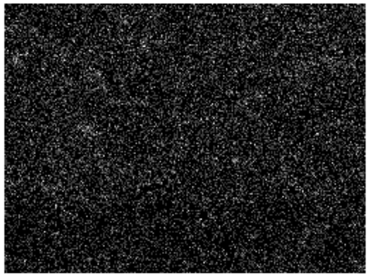

e. SEM-EDS map for S Ka; sample appears homogenous.

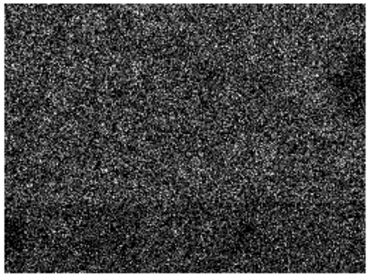

f. SEM-EDS map for Cl Ka; sample appears homogenous.

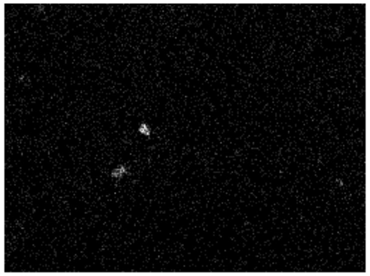

g. SEM-EDS map for Ca Ka; some calcium rich regions are visible on the surface. These correspond to surface features visible in the SEM image.

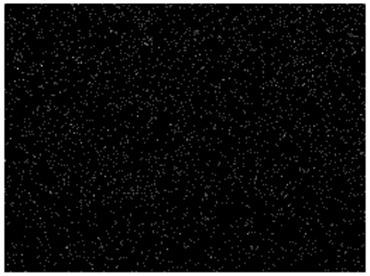

h. SEM-EDS map for Cu Ka; sample appears homogenous.

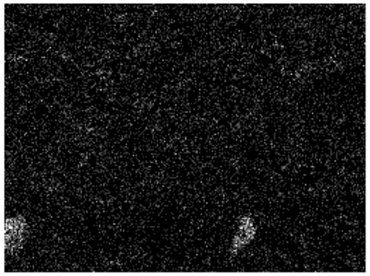

i. SEM-EDS map for Ba La; sample appears homogenous, except for a two regions that are more rich in barium. These correspond to isolated surface particulates that are visible in the SEM image.

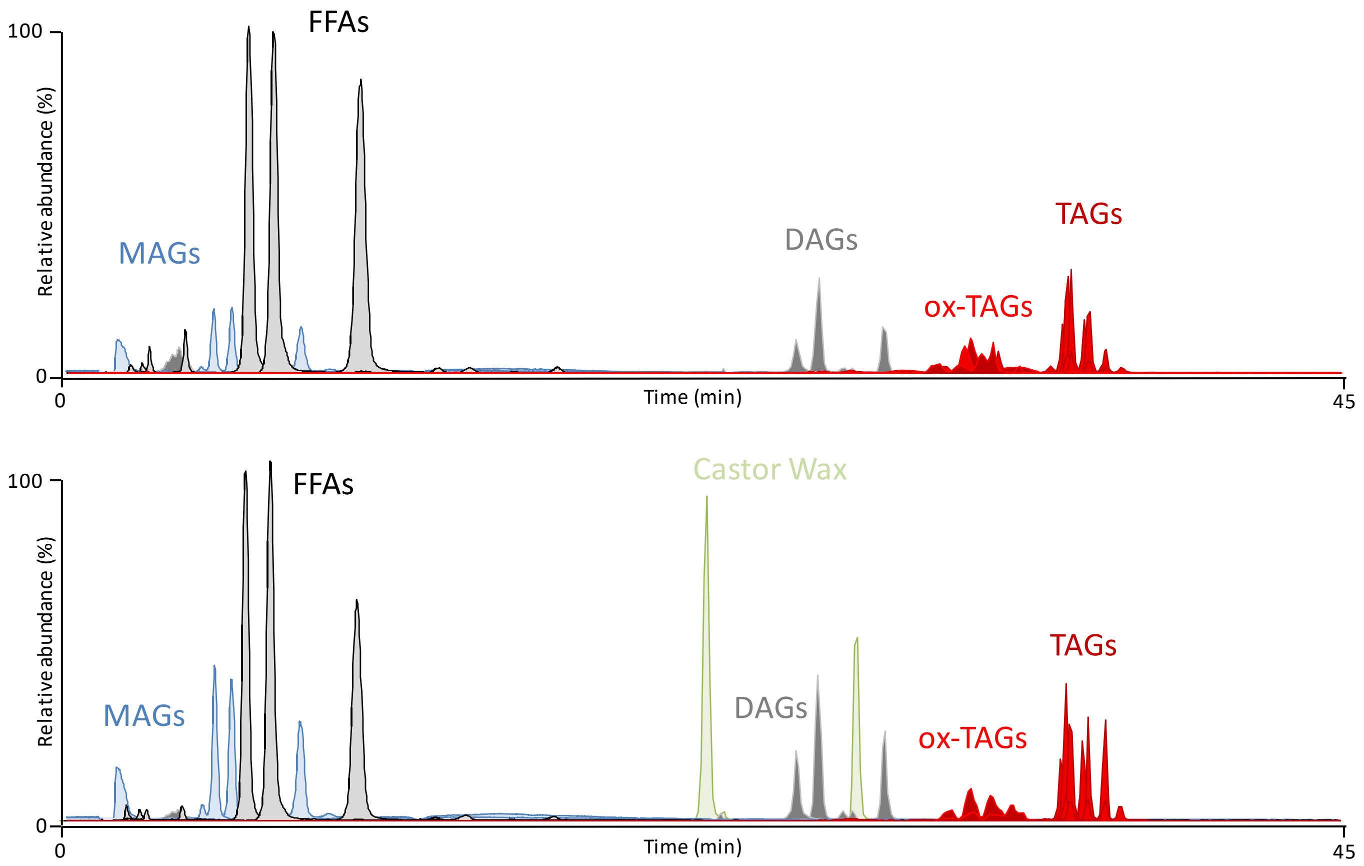

Figure S.5 HPLC extract ion chromatograms for NWS-1993 (top, non-water sensitive) and WS-2003 (bottom, water sensitive). FFA are detected as their HQ derivatives

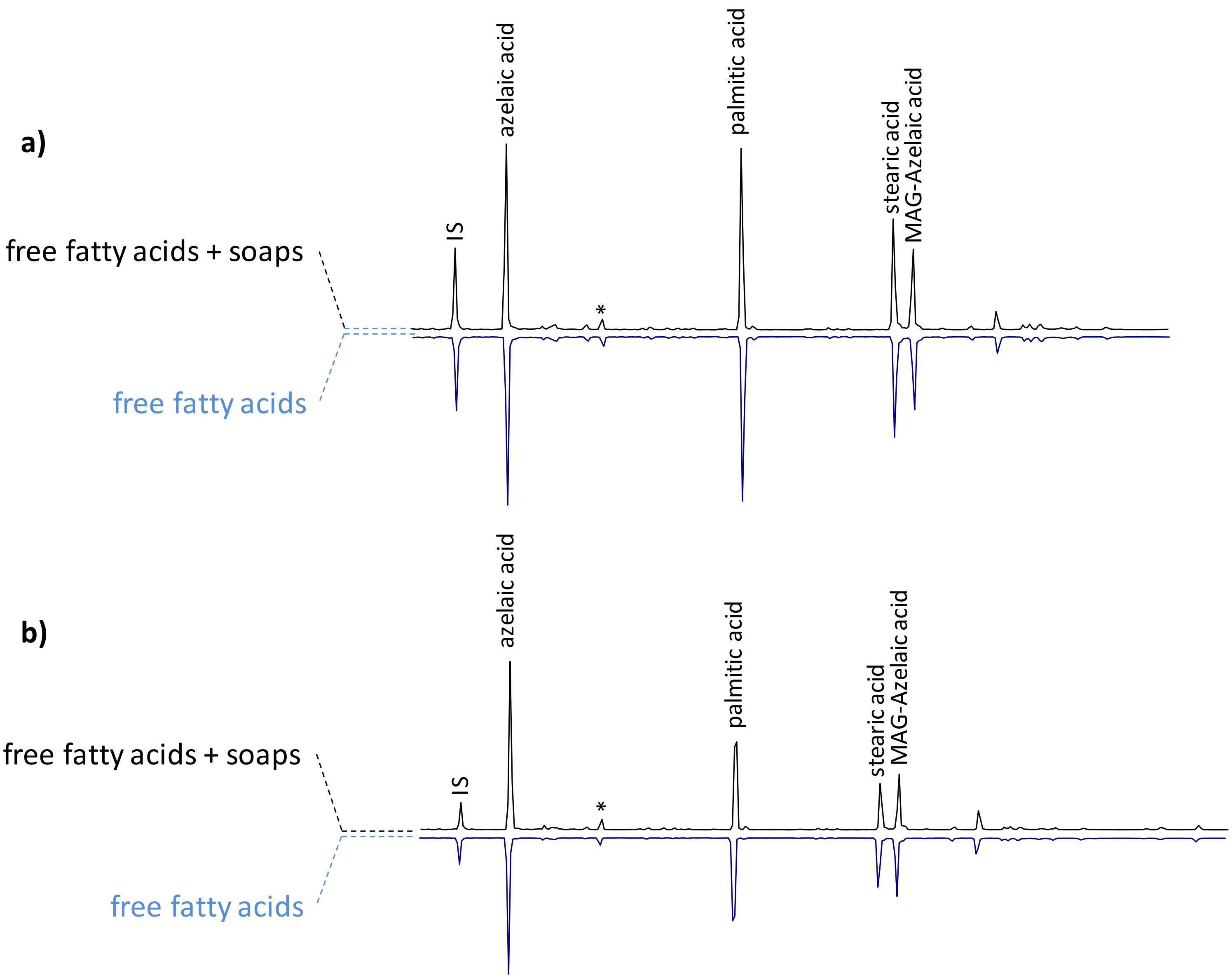

Figure S.6. GC-MS chromatograms obtained with the two-step derivatization procedures for a) WS-2003 b) NWS-1993

Figure S.7. TGA of phthalocyanine green (PG7) pigment provided by Windsor and Newton. A TA Instruments Thermobalance model Q5000 was used. TG measurement on the PG7 was performed at 20 °C/min scan rate, from 50 °C to 850°C under N_2 flow (25 mL/min).

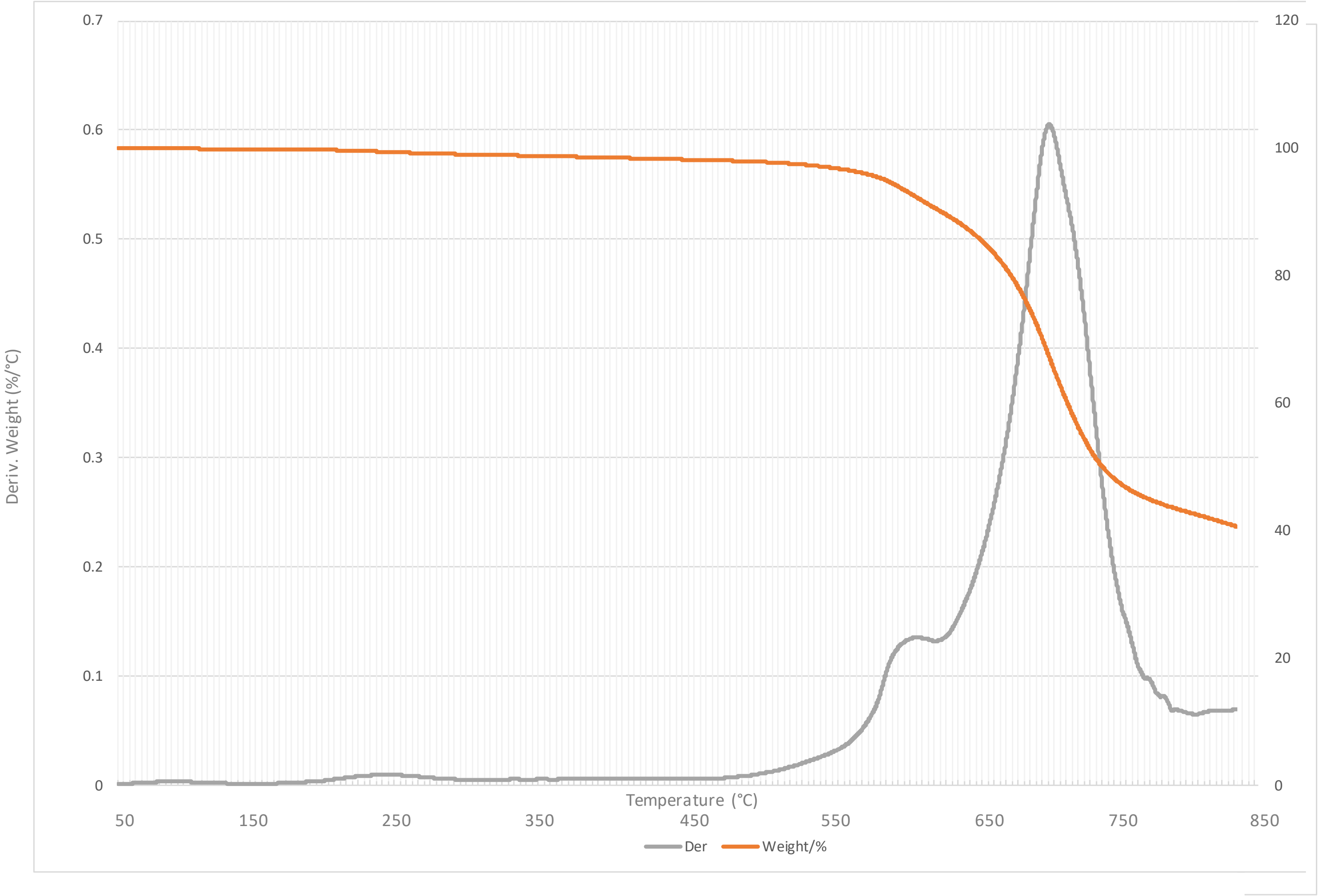

Figure S.8 – Mass spectrum obtained for the first thermal zone of NWS-1993

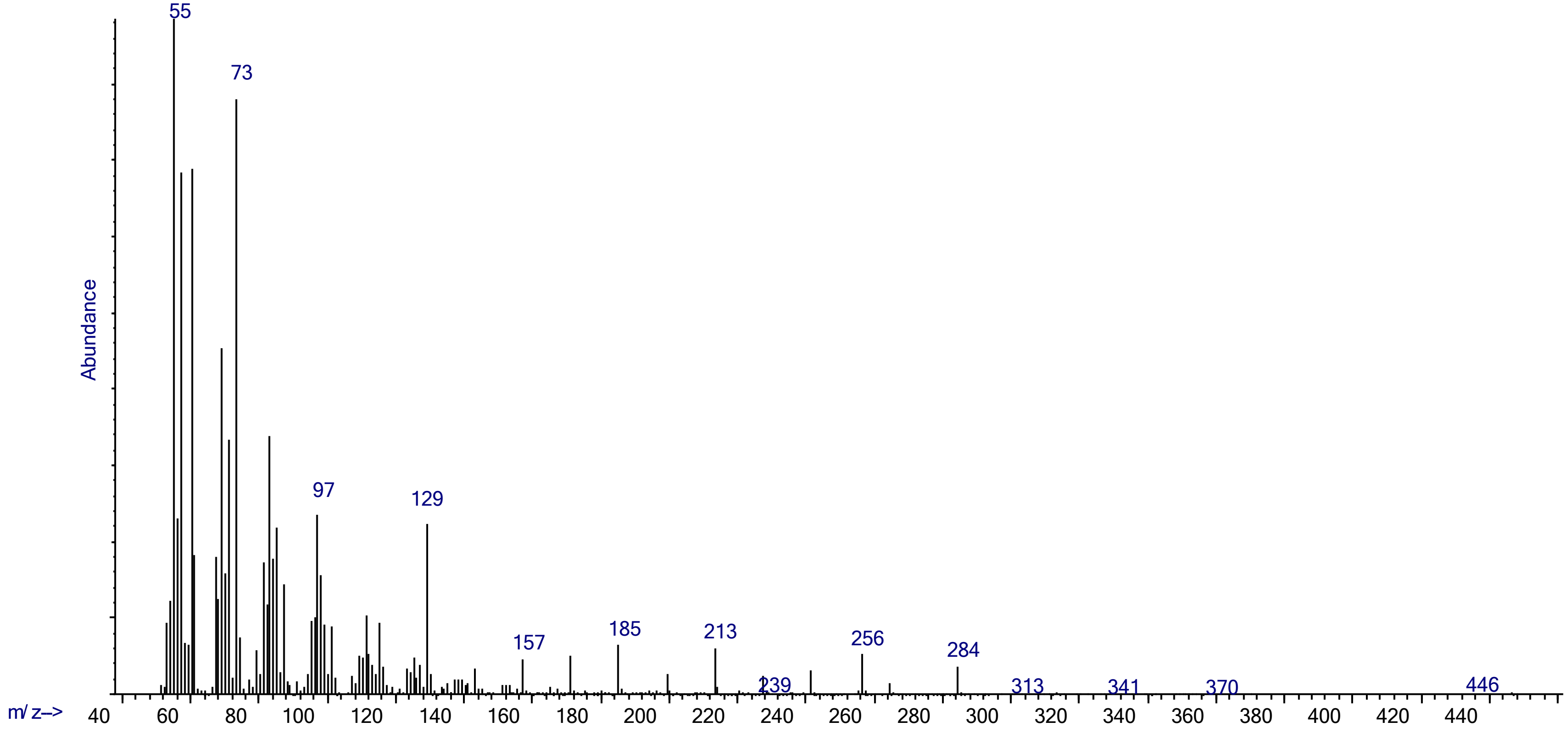

Figure S.9 – Mass spectrum obtained for the second thermal zone of NWS-1993

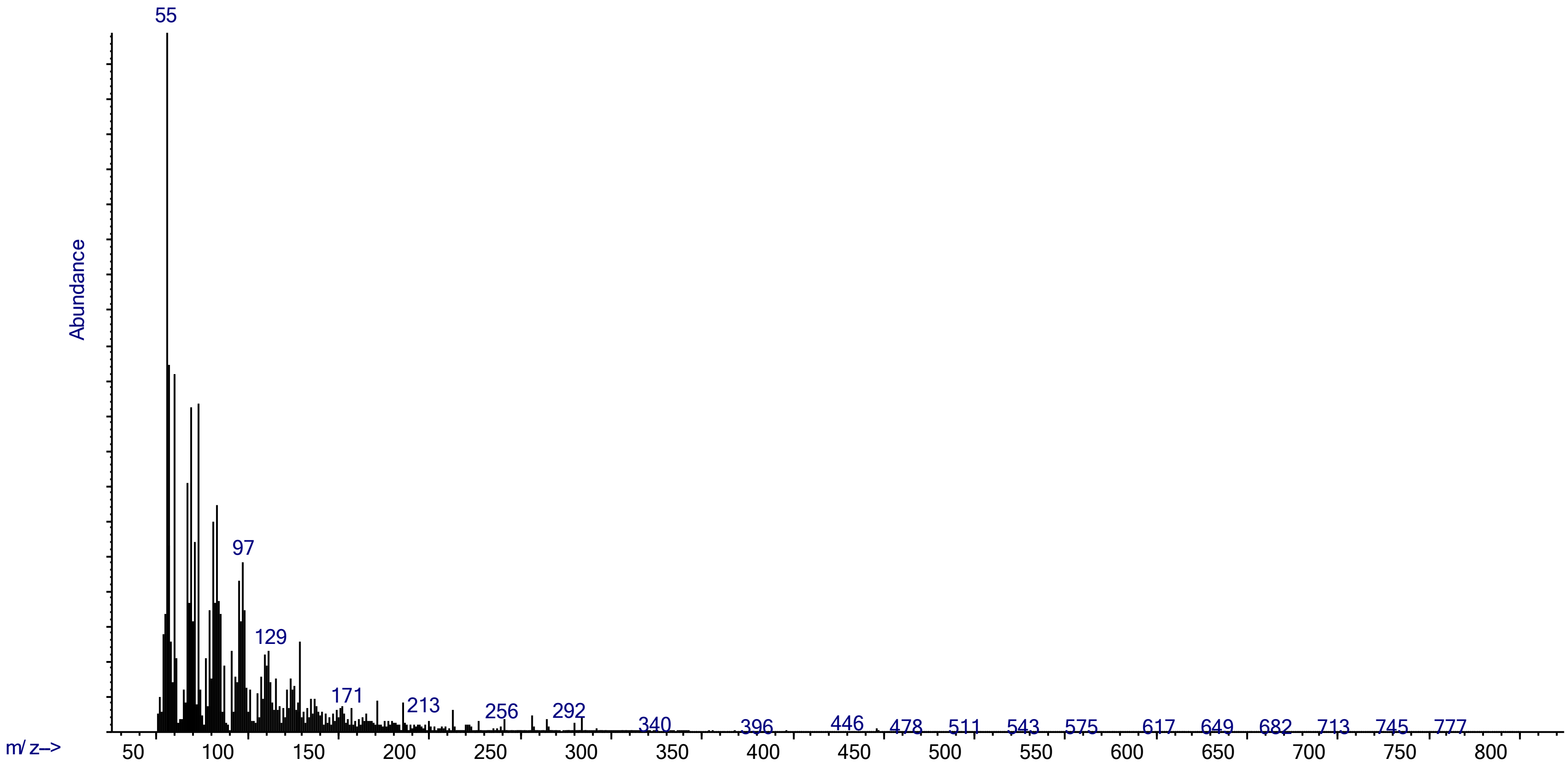

Figure S.10 – Mass spectrum obtained for the third thermal zone of NWS-1993

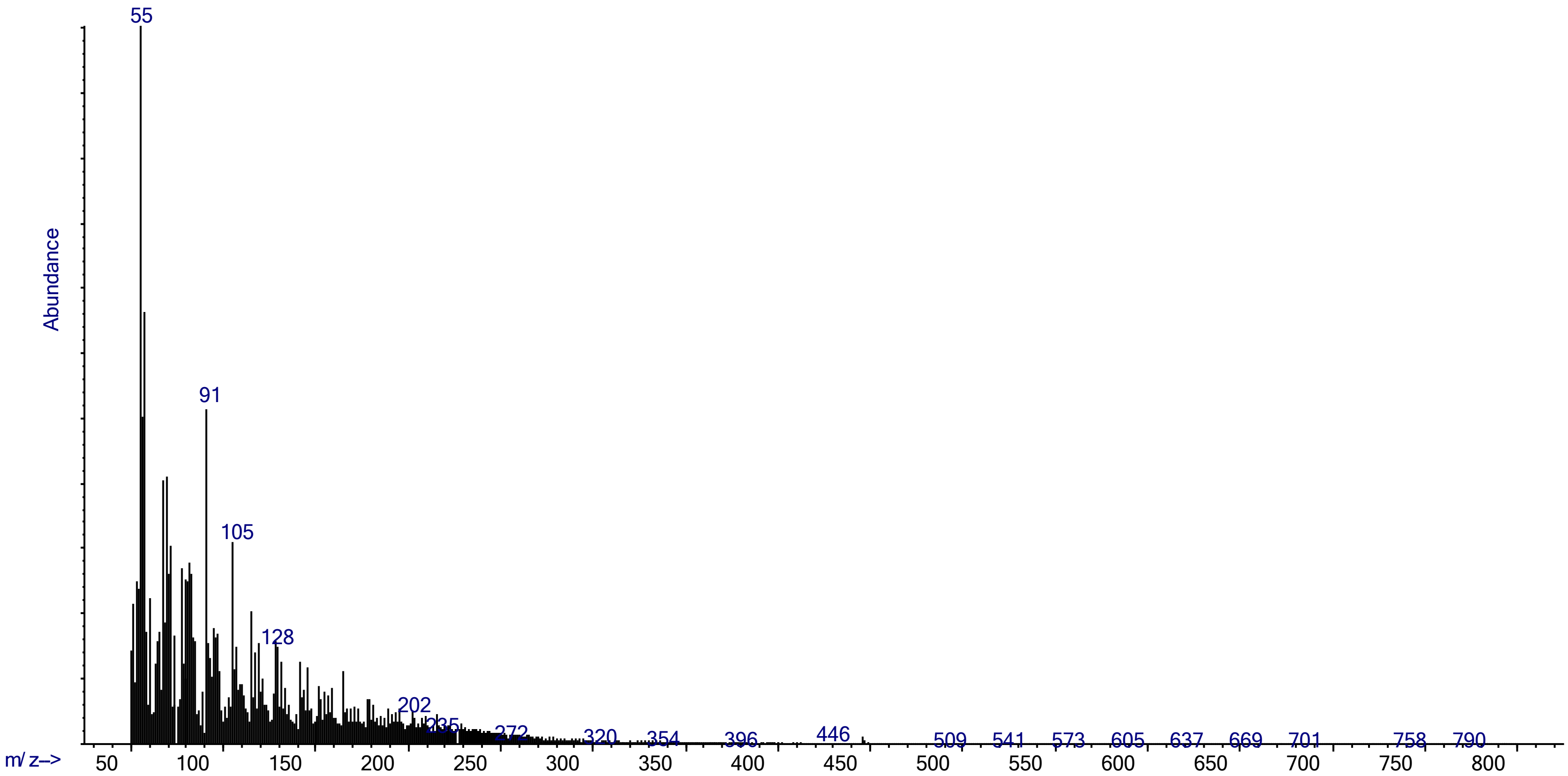

Figure S.11 – Mass spectrum obtained for the first thermal zone of NWS-2003

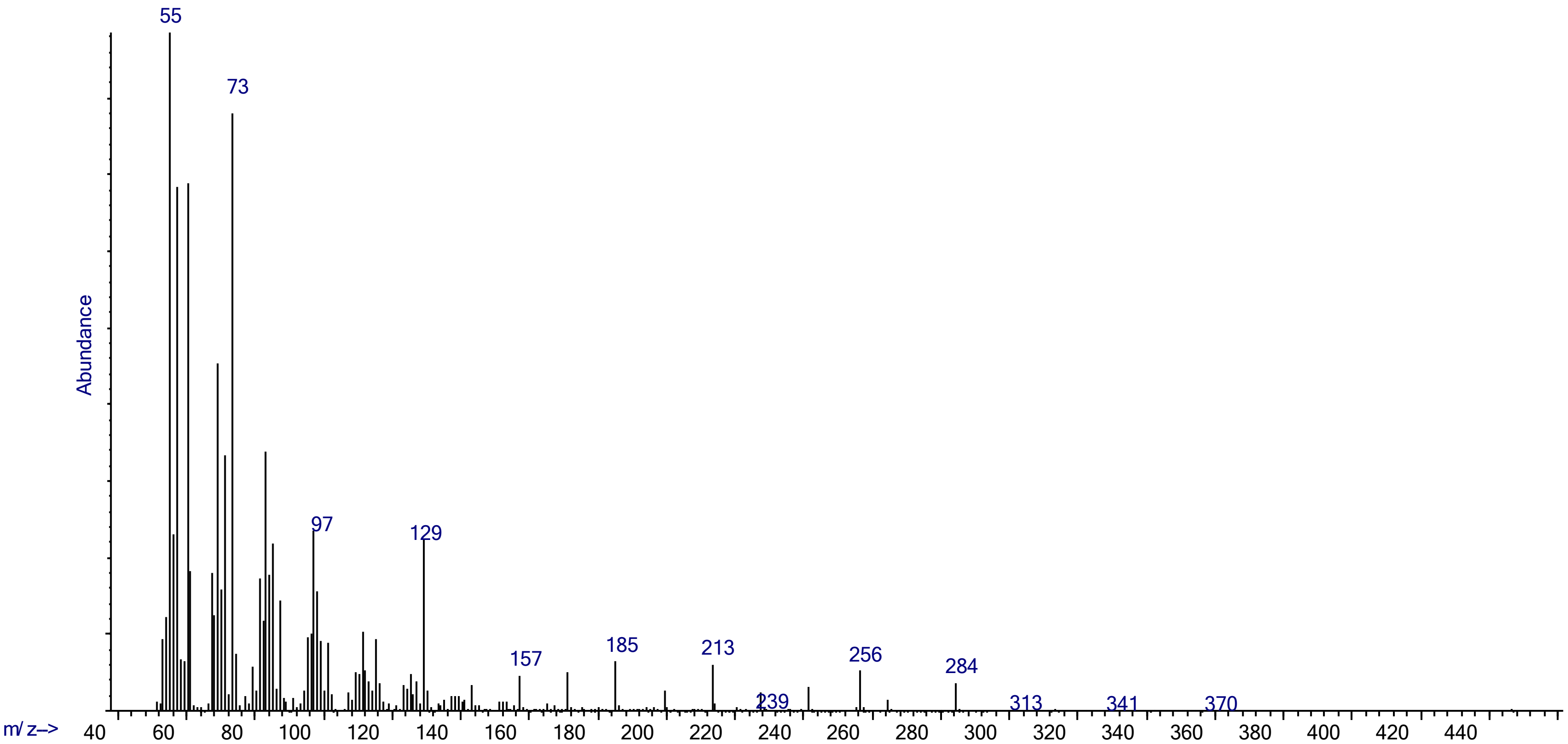

Figure S.12 – Mass spectrum obtained for the second thermal zone of NWS-2003

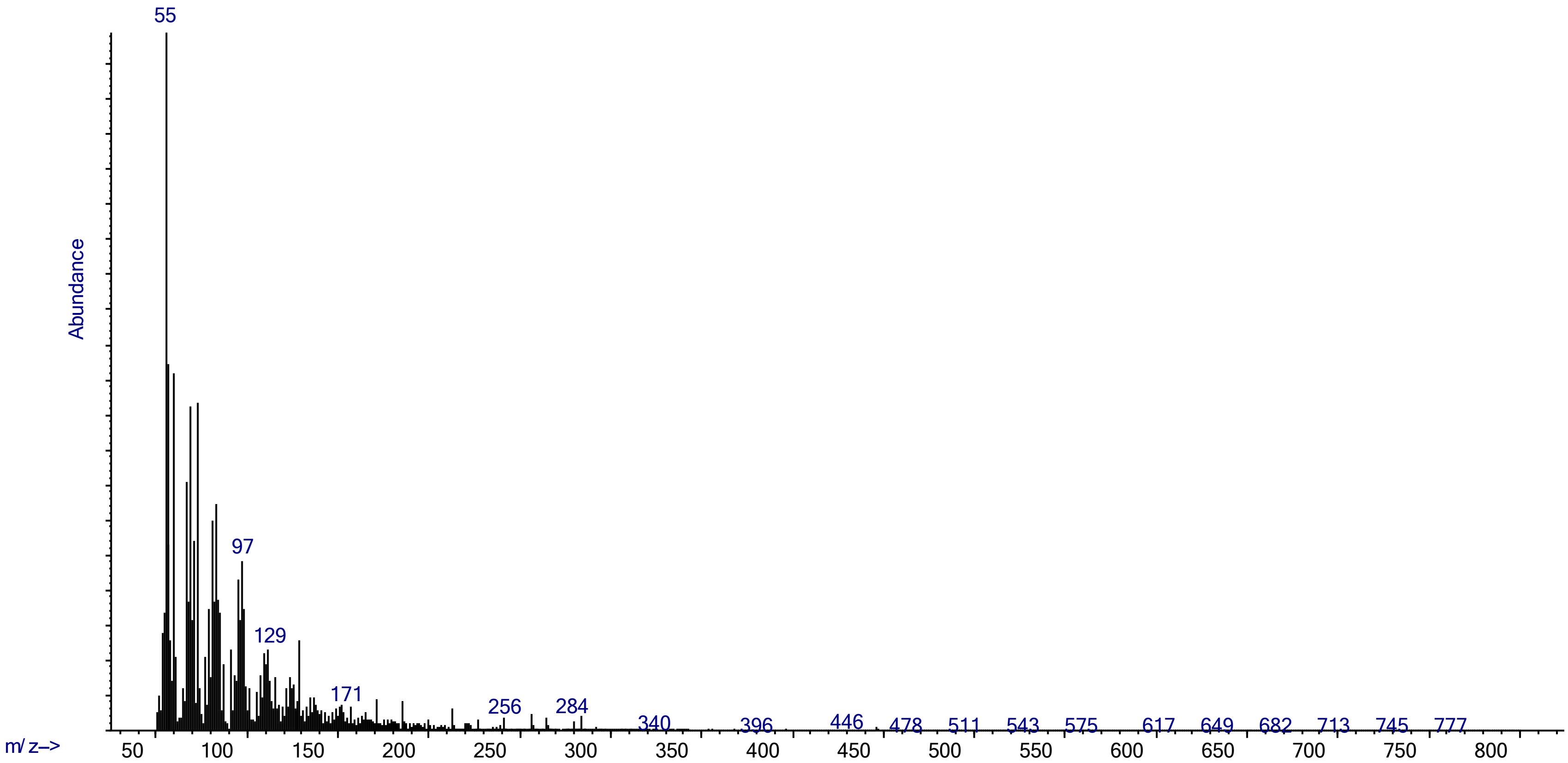

Figure S.13 – Mass spectrum obtained for the third thermal zone of NWS-2003

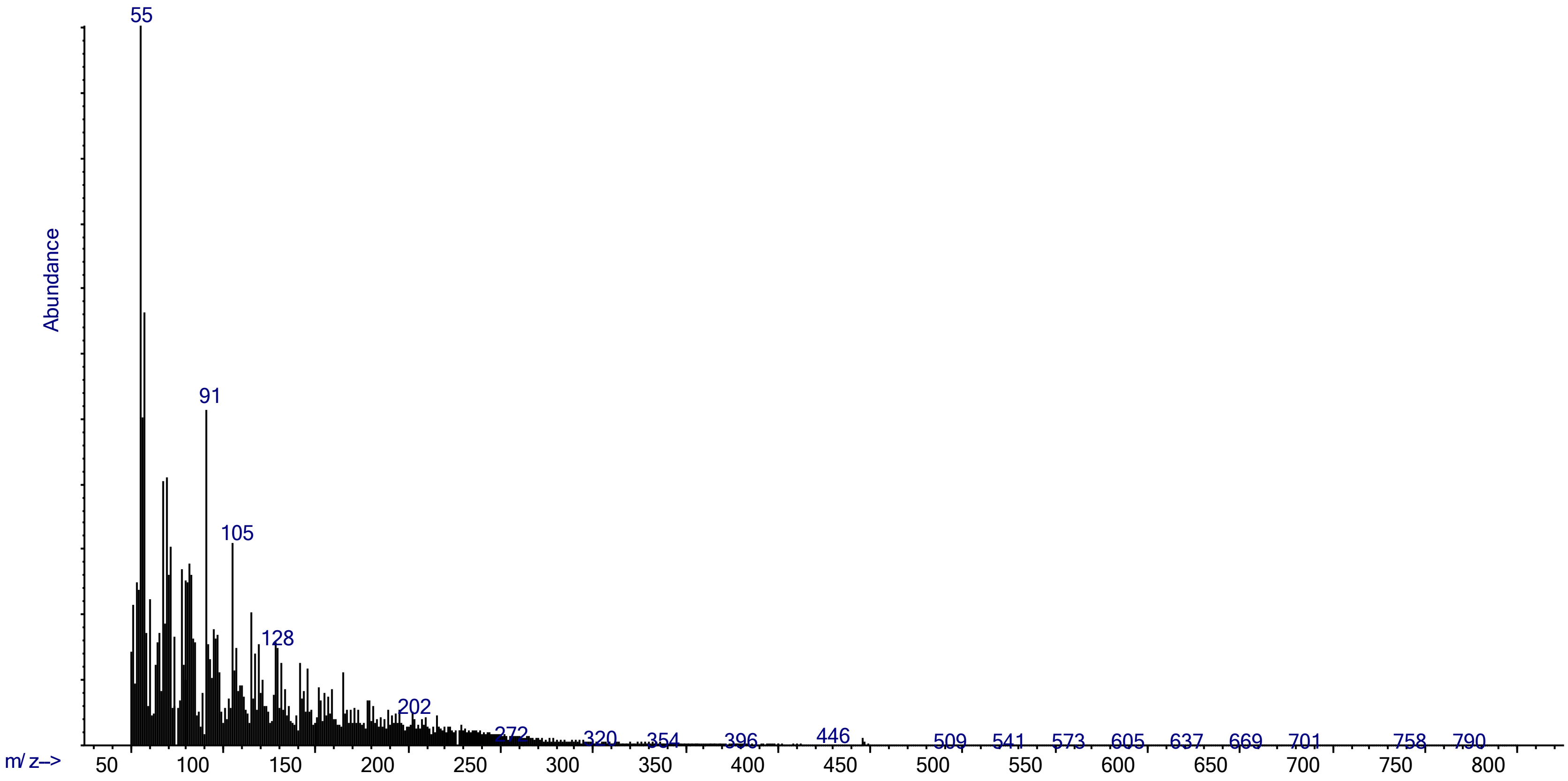

Table S.1 Relative abundances of fatty and dicarboxylic acids derived from GC-MS analysis

	Relative abundances (%)			
	1993		***2003***	
Fatty acid	HMDS	BSTFA	HMDS	BSTFA
Azelaic acid	33	32	21	22
Palmitic acid	23	23	28	21
Oleic acid	27	23	20	15
Stearic acid	17	15	22	14
